# Supplementary material for: Combinatorial effects of multiple genes contribute to beneficial aneuploidy phenotypes
Source: EMBO Rep. 2026 Apr 11;27(10):2772–97. doi: 10.1038/s44319-026-00767-8 (PMC13219433; doi:10.1038/s44319-026-00767-8)
Supplement: Supplementary file 1 — Appendix [file 44319_2026_767_MOESM1_ESM.pdf]

Appendix

Combinatorial effects of multiple genes contribute to beneficial aneuploidy phenotypes

Koller et al.

Table of Content

Appendix Figure S1	2
Appendix Figure S2	3
Appendix Figure S3	5
Appendix Figure S4	7
Appendix Table S1	8
Appendix Table S2	9
Appendix Table S3	15
Appendix Table S4	16

Appendix Figure S1

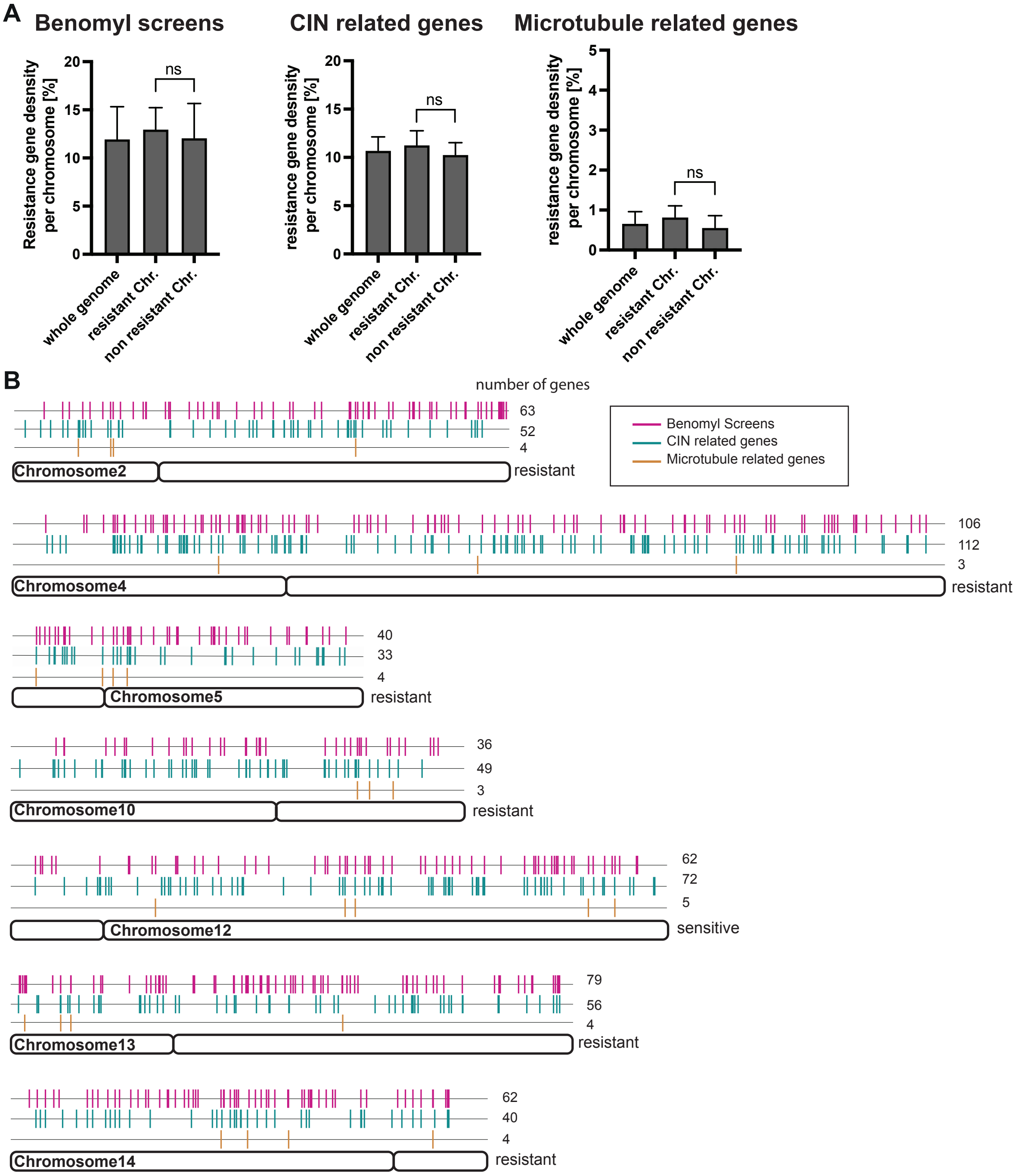

Appendix Figure S1

(A) Comparison of the distribution of candidate resistance genes based on different categories of GO terms between resistant chromosomes (2, 4, 5, 10, 13), non-resistant chromosomes, and all chromosomes (whole genome). Candidate resistance gene density was measured relative to all genes on a chromosome for each chromosome individually. Mean and standard deviation for each category are plotted. p-values are calculated using ordinary one-way ANOVA. (B) Distibution of candidate resistance genes for each category on benomyl resistant and sensitive chromosomes.

Appendix Figure S2

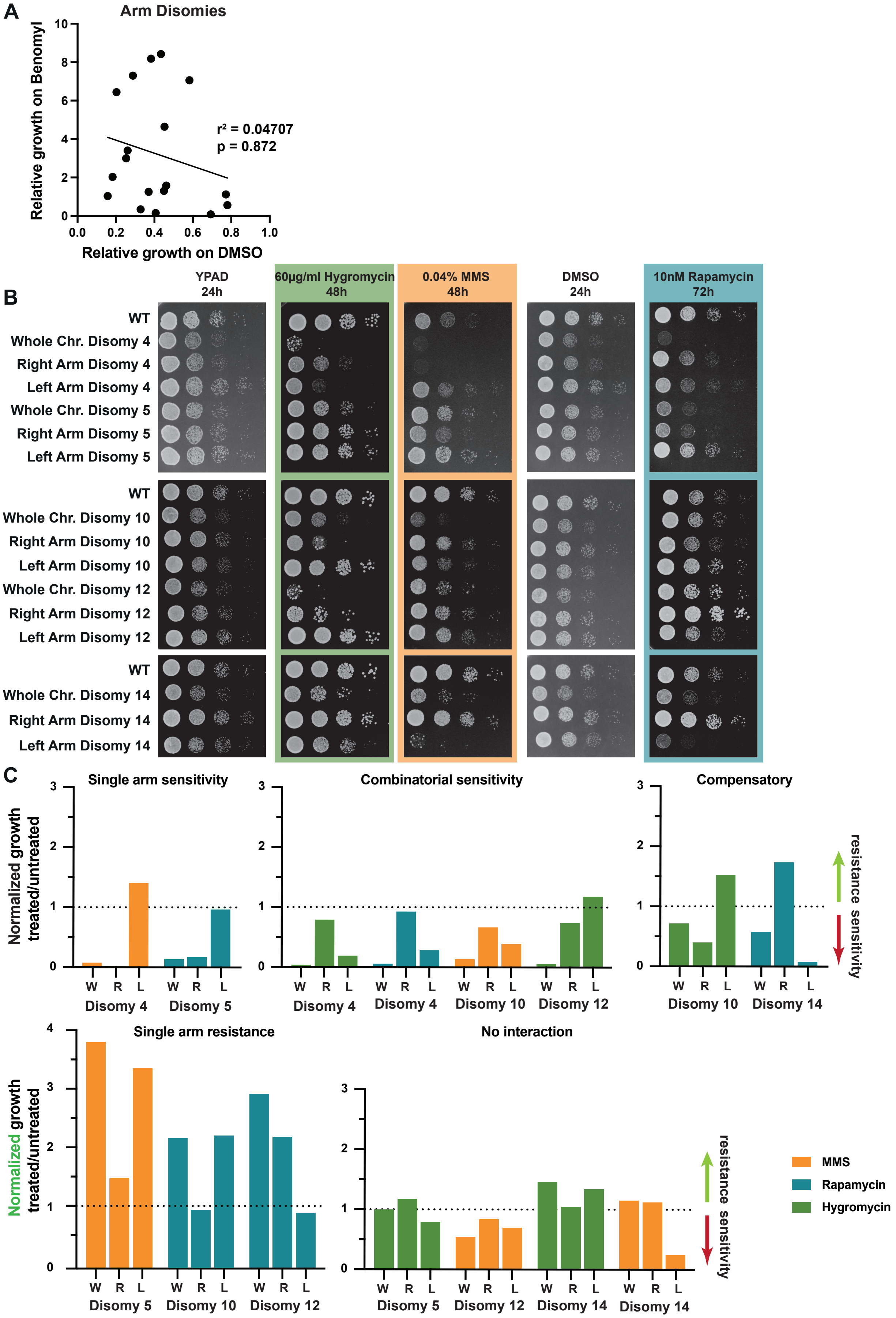

Appendix Figure S2

(A) Lack of correlation between growth of the arm level disomies on control (YPAD + DMSO) and treated plates (YPAD + 25 µg/ml benomyl) (r2 = 0.04707, p-value = 0.872). r^2 values are from simple linear regression and p-value are from *F*-tests. Quantification of Figure 4D. (B) 10-fold serial dilutions of whole chromosome and single arm aneuploidies on agar plates with YPAD, YPAD + DMSO, YPAD + 60 µg/ml hygromycin (green), YPAD + 0.04 % MMS (orange), or YPAD + 10 nM rapamycin (cyan). Timing of drug condition imaging was adjusted to the strength of the drug condition (hygromycin and MMS 48h, rapamycin 72h). (C) Quantifications of 10-fold serial dilutions from B. On control plates, the 3rd dilution was quantified and on treated plates, the 2nd dilution. Plate growth was first normalized to the haploid WT and then normalized to the untreated control. Each whole chromosome disomy (W) and the corresponding arm level disomies (L = left arm disomy, R = right arm disomy) was assigned to one of five categories: single arm sensitivity – one arm is treatment sensitive, combinatorial sensitive – both chromosome arms contribute to the whole chromosome sensitivity, compensatory sensitivity – a resistant arm can compensate for the sensitivity of a sensitive arm, single arm resistance – a single arm contributes to treatment resistance, and no interaction – whole chromosome disomy and single arm disomies are unaffected by the treatment.

Appendix Figure S3

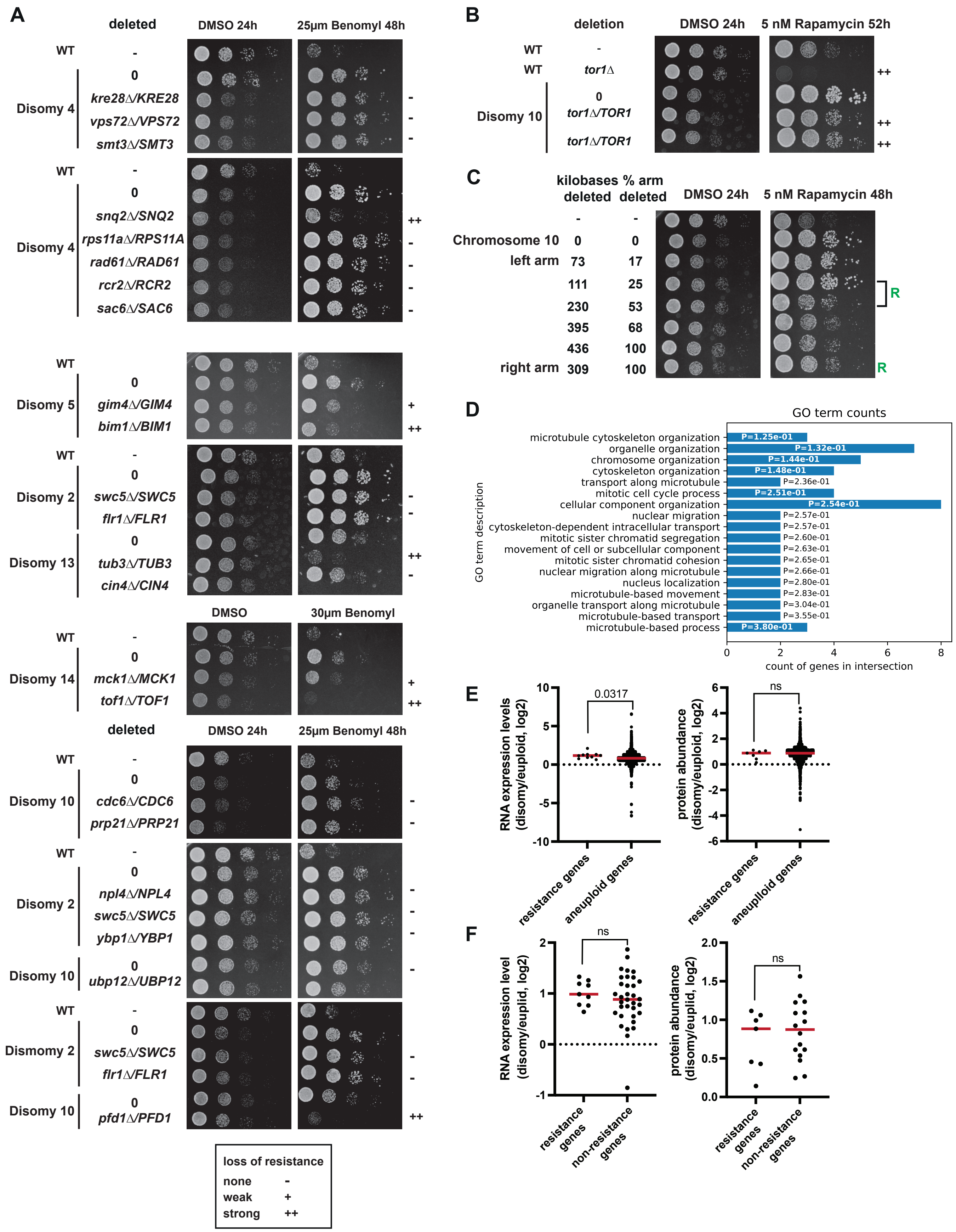

Appendix Figure S3

(A) 10-fold serial dilutions of candidate benomyl resistance genes. Phenotype classifications are annotated on the right and characterized as: none (-), weak (+), and strong (++). (B) 10-fold serial dilution of Δ*tor1* phenotypes on rapamycin. The phenotype classifications were annotated as for panel A. The 10-fold serial dilution of disomy 13, disomy 13 tub3Δ/TUB3, and disomy 13 cin4Δ/CIN4 on YPAD + DMSO (24h) and YPAD + 20 μg/ml benomyl (48h) plates are also shown in Figure EV3 (C) 10-fold serial dilutions of partial chromosome arm deletions of chromosome 10. Control plates (YPAD + DMSO) were imaged after 24h, and treated plates (YPAD + 5nM rapamycin) after 48h. Regions associated with resistance are indicated with an (R) on the right side. (D) Gene ontology over-representation analysis for identified benomyl resistance driver genes. Absolute FDR adjusted p-values (mHG model and Benjamini and Hochberg correction) are shown. (E) Expression level increase on aneuploid chromosomes for benomyl resistance genes compared to all genes on the aneuploid chromosomes. Means are indicated by red lines, p-values are from Mann-Whitney U tests. (F) Expression levels of identified benomyl resistance genes compared to the expression levels of candidate genes that didn't contribute to benomyl resistance. Means are indicated by red lines, p-values are from Mann-Whitney U tests. Transcriptome data are from Torres et al. 2007 (chromosomes 2, 4, 5, 10, 13, 14). Proteome data are from Dephoure et al. 2014 (chromosomes 2, 5, 10, 13, 14).

Appendix Figure S4

Chromosome		Region	Type of region	Gene
2	Cen2	0 - 237,423	none	
		238,979 - 341,042	sensitivity	*FLR1*
		341,042 - 436,902	none	
		436,902 - 537847	none	
		537,847 - 589,726	resistance	*CDC28, SLI15, APD1, NPL4*
		589,726 - 642,519	none	
		642,519 - 665,194	resistance	*YBP1, AME1*
		665,194 - 686,861	none	*SWC5, DAD3*
		686,861 - 713,057	none	
		713,057 - 737,873	none	
4	Cen4	0 - 449,243	sensitivity	
		449-859 - 497,791	resistance	***SNQ2,*** *RCR2, RPS11A, RAD61*
		497,791 - 598898	none	
		598,898 - 700,500	resistance	*ARP10, PDS1, DPB4, APC4*
		700,500 - 899,451	none	*SAC6*
		899,451 - 1,071,899	none	*CHL4*
		1,071,899 - 1,172,361	resistance	***SWR1***
		1,172,361 - 1,384,937	none	
		1,384,937 - 1,531,933	sensitivity	*EFT2, SPC110, ERD1, SMT3, LCD1, VPS72, KRE28*
5		0 - 76,019	none	
		76,019 - 133,182	mild resistance	
	Cen5	133,182 - 151,182	resistance	***GIM4***
		152,907 - 182,637	none	*PAC2*
		182,637 - 242,199	resistance	***BIM1****, GLN3*
		242,199 - 301,923	resistance	***SAP1***
		301,923 - 364,508	none	
		364,508 - 415,848	none	*MAM1*
		415,848 - 576,874	resistance	
10		0 - 72,703	mild resistance	*CDC6, PRP21, ECM25, UBP12*
		72,703 - 110,725	resistance	***PFD1***
		110,725 - 220,012	none	
		220,012 - 328,956	none	
	Cen10	328,956 - 435,981	sensitivity	*MAD3, MAD2*
		436,802 - 745,751	none	
12	Cen12	0 - 150,711	none	
		151,725 - 489,957	strong sensitvity	
		489,957 - 1,078,177	sensitivity	*TUB4*
13	Cen13	0 - 268,031	resistance	***TUB3****, TUB1*
		268,149 - 924,431	none	*CIN4*
14		0 - 120,998	mild resistance	***MCK1****, CLA4*
		120,998 - 220,164	resistance	***TOF1***
		220,164 - 320,127	none	
	Cen14	320,127 - 627,772	mild sensitivity	*ALF1*
		629,032 - 784,333	none	

Appendix Figure S4

Phenotypes for regions between all full and partial chromosome arm deletions engineered in this study. Centromeres are indicated by a dashed line. Phenotypes that were assigned to a region are annotated in 'Type of region'. Three types of phenotypes were assigned: none – no change in phenotype upon deletion, sensitivity – increased benomyl resistance upon deletion, mild sensitivity – mild decrease in benomyl resistance upon deletion, resistance – substantial decrease in in benomyl resistance upon deletion. All genes tested are listed; genes that decrease resistance when heterozygously deleted in the aneuploid strain are in bold.

Appendix Table S1. Overview of all strains represented in the chromosome gain collection

			URA3/HIS3																																													
			1	1	1	2	2	2	3	3**	3**	4	4	4	5	5	5	6	6	6	7	7	7	8	8	8	9	9	9	10	10	10	10	11	11	12	12	12	13	13	13	14	14	14	15	15	15	16	16	16
LYS2/LEU2	1	1				2x1	2x1	2x1	3x1	3x1	3x1	4x1	4x1	4x1	5x1	5x1	5x1	6x1	6x1	6x1	7x1	7x1	7x1	8x1	8x1	8x1	9x1	9x1	9x1	10x1	10x1	10x1	11x1	11x1	11x1	12x1	12x1	12x1	13x1	13x1	13x1	14x1	14x1	14x1	15x1	15x1	15x1	16x1	15x1	15x1
	2	2	1x2	1x2	1x2				3x2	3x2	3x2	4x2	4x2	4x2	5x2	5x2	5x2	6x2	6x2	6x2	7x2	7x2	7x2	8x2	8x2	8x2	9x2	9x2	9x2	10x2	10x2	10x2	11x2	11x2	11x2	12x2	12x2	12x2	13x2	13x2	13x2	14x2	14x2	14x2	15x2	15x2	15x2	16x2	15x2	15x2
	3	3**	1x3*	1x3*	1x3*	2x3	2x3	2x3				4x3	4x3	4x3	5x3	5x3	5x3	6x3	6x3	6x3	7x3	7x3	7x3	8x3	8x3	8x3	9x3	9x3	9x3	10x3	10x3	10x3	11x3	11x3	11x3	12x3	12x3	12x3	13x3	13x3	13x3	14x3	14x3	14x3	15x3	15x3	15x3	16x3	16x3	16x3
	4	4	1x4	1x4	1x4	2x4	2x4	2x4	3x4	3x4	3x4				5x4	5x4	5x4	6x4	6x4	6x4	7x4*	7x4	7x4	8x4	8x4	8x4	9x4	9x4	9x4	10x4	10x4	10x4*	11x4	11x4	11x4	12x4	12x4	12x4	13x4	13x4*	13x4*	14x4*	14x4	14x4	15x4	15x4	15x4	16x4	16x4	16x4
	5	5	1x5	1x5	1x5	2x5	2x5	2x5	3x5	3x5	3x5	4x5	4x5	4x5				6x5	6x5	6x5	7x5	7x5	7x5	8x5	8x5	8x5	9x5	9x5	9x5	10x5	10x5	10x5	11x5	11x5	11x5	12x5	12x5	12x5	13x5	13x5*	13x5*	14x5	14x5	14x5	15x5	15x5	15x5	16x5	16x5	16x5
	6	6	1x6	1x6	1x6	2x6	2x6	2x6	3x6	3x6	3x6	4x6	4x6	4x6	5x6	5x6	5x6				7x6	7x6	7x6	8x6	8x6	8x6	9x6	9x6	9x6	10x6	10x6	10x6	11x6	11x6	11x6	12x6	12x6	12x6	13x6	13x6	13x6	14x6	14x6	14x6	15x6	15x6	15x6	16x6	16x6	16x6
	7	7	1x7	1x7	1x7	2x7	2x7	2x7	3x7	3x7	3x7	4x7	4x7	4x7	5x7	5x7	5x7	6x7	6x7	6x7				8x7	8x7	8x7	9x7	9x7	9x7	10x7	10x7	10x7	11x7	11x7	11x7	12x7	12x7	12x7	13x7	13x7	13x7	14x7*	14x7	14x7	15x7	15x7	15x7	16x7	16x7	16x7
	8	8	1x8	1x8	1x8*	2x8	2x8	2x8	3x8	3x8	3x8	4x8	4x8	4x8	5x8	5x8	5x8	6x8	6x8	6x8	7x8	7x8	7x8				9x8	9x8	9x8	10x8	10x8	10x8	11x8	11x8	11x8	12x8	12x8	12x8	13x8	13x8	13x8	14x8	14x8	14x8	15x8	15x8	15x8	16x8	16x8	16x8
	9	9	1x9	1x9	1x9	2x9	2x9	2x9	3x9	3x9	3x9	4x9	4x9	4x9	5x9	5x9	5x9	6x9	6x9	6x9	7x9	7x9	7x9	8x9	8x9	8x9				10x9	10x9	10x9	11x9	11x9	11x9	12x9	12x9	12x9	13x9	13x9	13x9	14x9	14x9	14x9	15x9	15x9	15x9	16x9	16x9	16x9
	10	10	1x10	1x10	1x10	2x10	2x10	2x10	3x10	3x10	3x10	4x10	4x10	4x10*	5x10	5x10	5x10	6x10	6x10	6x10	7x10	7x10	7x10	8x10	8x10	8x10	9x10	9x10	9x10				11x10	11x10	11x10	12x10	12x10	12x10	13x10	13x10	13x10*	14x10*	14x10	14x10	15x10	15x10	15x10	16x10	16x10	16x10*
	11	11	1x11	1x11	1x11	2x11	2x11	2x11	3x11	3x11	3x11	4x11	4x11	4x11*	5x11	5x11	5x11	6x11	6x11	6x11	7x11	7x11	7x11	8x11	8x11	8x11	9x11	9x11	9x11	10x11	10x11	10x11				12x11	12x11	12x11*	13x11	13x11	13x11	14x11	14x11	14x11*	15x11	15x11	15x11	16x11*	16x11*	16x11*
	12	12	1x12	1x12	1x12	2x12	2x12	2x12	3x12	3x12	3x12	4x12	4x12	4x12	5x12	5x12	5x12	6x12	6x12	6x12	7x12	7x12	7x12	8x12*	8x12	8x12	9x12	9x12	9x12	10x12	10x12	10x12	11x12	11x12*	11x12*				13x12*	13x12*	13x12*	14x12	14x12	14x12	15x12	15x12	15x12	16x12	16x12	16x12
	13	13	1x13	1x13	1x13	2x13	2x13	2x13	3x13	3x13	3x13	4x13	4x13	4x13	5x13	5x13	5x13	6x13	6x13	6x13	7x13	7x13	7x13	8x13*	8x13	8x13	9x13	9x13	9x13	10x13	10x13	10x13	11x13	11x13	11x13	12x13	12x13	12x13				14x13	14x13*	14x13	15x13	15x13	15x13	16x13	16x13	16x13*
	14	14	1x14	1x14	1x14	2x14*	2x14	2x14	3x14	3x14	3x14	4x14	4x14	4x14	5x14	5x14	5x14	6x14	6x14	6x14	7x14	7x14*	7x14	8x14	8x14	8x14	9x14	9x14	9x14	10x14	10x14	10x14	11x14	11x14	11x14	12x14	12x14	12x14	13x14*	13x14*	13x14*				15x14	15x14	15x14	16x14	16x14	16x14
	15	15	1x15	1x15	1x15	2x15	2x15	2x15	3x15	3x15	3x15	4x15	4x15	4x15	5x15	5x15	5x15	6x15	6x15	6x15	7x15	7x15	7x15	8x15	8x15	8x15	9x15	9x15	9x15	10x15	10x15	10x15	11x15	11x15	11x15	12x15	12x15	12x15	13x15	13x15	13x15	14x15	14x15	14x15				16x15	16x15	16x15
	16	16	1x16	1x16	1x16	2x16	2x16	2x16	3x16	3x16	3x16	4x16	4x16	4x16	5x16	5x16	5x16	6x16	6x16	6x16	7x16	7x16	7x16	8x16	8x16	8x16	9x16	9x16	9x16	10x16	10x16	10x16	11x16*	11x16*	11x16*	12x16	12x16	12x16	13x16*	13x16*	13x16*	14x16	14x16	14x16	15x16	15x16	15x16			

*diploid / mixed by FACS - excluded from the analysis

*** qPCR revealed that strains had gained 3 copies of a chromosome and where therefore excluded from the analysis.* gained 3 copies of a chromosome

Appendix Table S2. Strains used in this study

Strain	Genotype / Description	Source	Background	Figures
BY4741	*MATa, his3Δ1, leu2Δ0, ura3Δ0, met15Δ0*	a	S288c	-
BY4742	*MATα, his3Δ1, leu2Δ0, ura3Δ0*	a	S288c	-
CCY1914	*MATa/MATα, his3Δ1/his3Δ1, leu2Δ0/leu2Δ0, ura3Δ0/ ura3Δ0, lys2D0/lys2D0*	b	S288c	1a
CCY2092	*his3D1/his3D1; leu2D0/leu2D0; lys2D0/lys2D0; ura3D2/ura3D2; cen13::p-GAL1-CEN3::URA3*	b	S288c	1b, d, EV1c
CCY3146	*his3D1/his3D1; leu2D0/leu2D0; lys2D0/lys2D0; ura3D2/ura3D2; cen13::p-GAL1-CEN3::URA3, tub2Δ::HYGRO*	b	S288c	1d
LL 6x13*	*his3D1/HIS3; leu2D0/LEU2; lys2D0/lys2D0; ura3D2/ura3D2; cen13::p-GAL1-CEN3::URA3, cen6::p-GAL1-CEN3::URA3*	b		1b, d, EV1d
CCY3837	*MATa/α his3D1/HIS3, leu2D0/LEU2, lys2D0/LYS2, met15D0/MET15, ura3D00/URA3*	b	S288c	-
CCY5419	*MATα, ura3Δ0, pRS316*	b	S288c	haploid wt in all figures
CCY5828	*MATα, ura3Δ0, pRS316, cen2::p-GAL1-CEN3:LYS2/cen2::p-GAL1- c CEN3:lys2::pCC644:LEU2*	b	S288c	1, 2, 3a,b, c, 4 d, e, 5c, EV1, EV2, EV3a, b, c, 5a, 7a,S4a
CCY5830	*MATα, ura3Δ0, pRS316, cen3::p-GAL1-CEN3:LYS2/cen3::p-GAL1- c CEN3:lys2::pCC644:LEU2*	b	S288c	1, 2, 3b, c, EV1, EV2, EV3a, b, c,
CCY5831	*MATα, ura3Δ0, pRS316, cen4::p-GAL1-CEN3:LYS2/cen4::p-GAL1- c CEN3:lys2::pCC644:LEU2*	b	S288c	1, 2, 3a, b, c, 4c, d, e, 5a, b, c, 6a, c, d, EV1, EV2, EV3a, b, c, S2a, b, c, EV4a, S3a, EV5a, b
CCY5833	*MATα, ura3Δ0, pRS316, cen5::p-GAL1-CEN3:LYS2/cen5::p-GAL1- c CEN3:lys2::pCC644:LEU2*	b	S288c	1, 2, 3a, b, c, 4c, d, e 5c, 6a, c, d, EV1, EV2, EV3a, b, c, S2a, b, c, EV4a, b, S3a, EV5a, b

CCY5835	*MATα, ura3Δ0, pRS316, cen7::p-GAL1-CEN3:LYS2/cen7::p-GAL1- c CEN3:lys2::pCC644:LEU2*	b	S288c	1, 2, 3b, c, EV1, EV2, EV3a, b, c
CCY5837	*MATα, ura3Δ0, pRS316, cen8::p-GAL1-CEN3:LYS2/cen8::p-GAL1- c CEN3:lys2::pCC644:LEU2*	b	S288c	1, 2, 3b, c, EV1, EV2, EV3a, b, c
CCY5839	*MATα, ura3Δ0, pRS316, cen9::p-GAL1-CEN3:LYS2/cen9::p-GAL1- c CEN3:lys2::pCC644:LEU2*	b	S288c	1, 2, 3b, c, EV1, EV2, EV3a, b, c
CCY5841	*MATα, ura3Δ0, pRS316, cen10::p-GAL1-CEN3:LYS2/cen10::p-GAL1- c CEN3:lys2::pCC644:LEU2*	b	S288c	1, 2, 3 a,b, c, 4d, e, 5c, 6a, c, d, EV1, EV2, EV3a, b, c, S2a, b, c, EV4a, b, S3a, b, EV9a, b
CCY5843	*MATα, ura3Δ0, pRS316, cen11::p-GAL1-CEN3:LYS2/cen11::p-GAL1- c CEN3:lys2::pCC644:LEU2*	b	S288c	1, 2, 3b, c, EV1, EV2, EV3a, b, c
CCY5845	*MATα, ura3Δ0, pRS316, cen12::p-GAL1-CEN3:LYS2/cen12::p-GAL1- c CEN3:lys2::pCC644:LEU2*	b	S288c	1, 2, 3b, c, EV1, EV2, EV3a, b, c, S2a, b, c, EV4a, EV5a, b, c
CCY5847	*MATα, ura3Δ0, pRS316, cen13::p-GAL1-CEN3:LYS2/cen13::p-GAL1- c CEN3:lys2::pCC644:LEU2*	b	S288c	1, 2, 3a, b, c, EV1, EV2, EV3a, b, c, d, S3a, EV9a, b
CCY5848	*MATα, ura3Δ0, pRS316, cen14::p-GAL1-CEN3:LYS2/cen14::p-GAL1- c CEN3:lys2::pCC644:LEU2*	b	S288c	1, 2, 3a, b, c, 4 d, e, 5c, 6a, c, d, EV1, EV2, EV3a, b, c, S2a, b, c, EV4a, S3a
CCY5849	*MATα, ura3Δ0, pRS316, cen16::p-GAL1-CEN3:LYS2/cen16::p-GAL1- c CEN3:lys2::pCC644:LEU2*	b	S288c	1, 2, 3b, c, EV1, EV2, EV3a, b, c
CCY5851	*MATa, LEU2, LYS2, cen1::p-GAL1-CEN3:URA3/cen1::p-GAL1- c CEN3:ura3::pCC631:HIS3*	b	S288c	1, 2, 3b, c, EV1, EV2, EV3a, b, c
CCY5854	*MATa, LEU2, LYS2, cen2::p-GAL1-CEN3:URA3/cen2::p-GAL1- c CEN3:ura3::pCC631:HIS3*	b	S288c	1, 2, 6a,c, EV1, EV2, EV5a

CCY5857	*MATa, LEU2, LYS2, cen3::p-GAL1-CEN3:URA3/cen3::p-GAL1- c CEN3:ura3::pCC631:HIS3*	b	S288c	1, 2, EV1, EV2, EV5c
CCY5858	*MATa, LEU2, LYS2, cen4::p-GAL1-CEN3:URA3/cen4::p-GAL1- c CEN3:ura3::pCC631:HIS3*	b	S288c	1, 2, EV1, EV2
CCY5864	*MATa, LEU2, LYS2, cen7::p-GAL1-CEN3:URA3/cen7::p-GAL1- c CEN3:ura3::pCC631:HIS3*	b	S288c	1, 2, EV1, EV2
CCY5866	*MATa, LEU2, LYS2, cen8::p-GAL1-CEN3:URA3/cen8::p-GAL1- c CEN3:ura3::pCC631:HIS3*	b	S288c	1, 2, EV1, EV2
CCY5869	*MATa, LEU2, LYS2, cen9::p-GAL1-CEN3:URA3/cen9::p-GAL1- c CEN3:ura3::pCC631:HIS3*	b	S288c	1, 2, EV1, EV2
CCY5872	*MATa, LEU2, LYS2, cen10::p-GAL1-CEN3:URA3/cen10::p-GAL1- c CEN3:ura3::pCC631:HIS3*	b	S288c	1, 2, EV1, EV2
CCY5875	*MATa, LEU2, LYS2, cen11::p-GAL1-CEN3:URA3/cen11::p-GAL1- c CEN3:ura3::pCC631:HIS3*	b	S288c	1, 2, EV1, EV2
CCY5878	*MATa, LEU2, LYS2, cen12::p-GAL1-CEN3:URA3/cen12::p-GAL1- c CEN3:ura3::pCC631:HIS3*	b	S288c	1, 2, EV1, EV2
CCY5881	*MATa, LEU2, LYS2, cen13::p-GAL1-CEN3:URA3/cen13::p-GAL1- c CEN3:ura3::pCC631:HIS3*	b	S288c	1, 2, EV1, EV2
CCY5884	*MATa, LEU2, LYS2, cen14::p-GAL1-CEN3:URA3/cen14::p-GAL1- c CEN3:ura3::pCC631:HIS3*	b	S288c	1, 2, EV1, EV2, EV5b
CCY5886	*MATa, LEU2, LYS2, cen16::p-GAL1-CEN3:URA3/cen16::p-GAL1- c CEN3:ura3::pCC631:HIS3*	b	S288c	1, 2, EV1, EV2
CCY6982 / C	*MATα, cen14::p-GAL1-CEN3:URA3/cen14::p-GAL1- c CEN3:ura3::pCC631:HIS3, cen10::p-GAL1-CEN3:LYS2/cen10::p-GAL1- c CEN3:lys2::pCC644:LEU2*	b	S288c	1, 2, EV1, EV2
CCY6576	*MATα,HIS3, leu2D0, lys2D0, URA3, cen2::p-GAL1-CEN3:LYS2/cen2::p-GAL1- c CEN3:lys2::pCC644:LEU2, Chr2L-237,423 bp::hphNT1::TEL*	b	S288c	4d, 6d
CCY6579	*CCY5828 + Chr2R-238,979 bp::hphNT1::TEL*	b	S288c	4d, EV4a, 6d
CCY7132	*CCY5828 + Chr2R-642,519 bp::hphNT1::TEL*	b	S288c	EV4a
CCY7134	*CCY5828 + Chr2R-537,847 bp::hphNT1::TEL*	b	S288c	EV4a
CCY7135	*CCY5828 + Chr2R-436,902 bp::hphNT1::TEL*	b	S288c	EV4a
CCY7137	*CCY5828 + Chr2R-341,042 bp::hphNT1::TEL*	b	S288c	EV4a
CCY7139	*CCY5828 + Chr2R-686,861 bp::hphNT1::TEL*	b	S288c	EV4a
CCY7140	*CCY5828 + Chr2R-589,726 bp::hphNT1::TEL*	b	S288c	EV4a
CCY7141	*CCY5828 + Chr2R-665,194 bp::hphNT1::TEL*	b	S288c	EV4a
CCY7143	*CCY5828 + Chr2R-713,057 bp::hphNT1::TEL*	b	S288c	EV4a
CCY6366	*CCY5828 + SWC5/swc5Δ::KanMX4*	b	S288c	S3a

CCY7101	*CCY5828 + FLR1/flr1Δ::hphNT1*	b	S288c	S3a
CCY6339	*CCY5831 + Chr4R-1,172,361 bp::KanMX4::TEL*	b	S288c	EV4a
CCY6343	*CCY5831 + Chr4R-1,071,899 bp::KanMX4::TEL*	b	S288c	5a, b
CCY6345	*CCY5831 + Chr4R-899,451 bp::KanMX4::TEL*	b	S288c	5a
CCY6348	*CCY5831 + Chr4R-700,500 bp::KanMX4::TEL*	b	S288c	5a
CCY6352	*CCY5831 + Chr4R-497,791 bp::KanMX4::TEL*	b	S288c	5a, b
CCY6355	*CCY5831 + Chr4R-449,859 bp::KanMX4::TEL*	b	S288c	4c, d, e, 5a, b, 6d, S2
CCY6357	*CCY5831 + Chr4L-449,243 bp::KanMX4::TEL*	b	S288c	4c, d, e, 5a, b, 6d, S2
CCY6360	*CCY5831 + Chr4R-1,384,937 bp::KanMX4::TEL*	b	S288c	5a
CCY6361	*CCY5831 + Chr4R-598,898 bp::KanMX4::TEL*	b	S288c	5a
CCY6369	*CCY5831 + SWR1/swr1Δ::KanMX4*	b	S288c	5b
CCY6373	*CCY5831 + SNQ2/snq2Δ::hphNT1*	b	S288c	5b, EV4a
CCY6375	*CCY5831 + RPS11A/rps11aΔ::hphNT1*	b	S288c	5b, EV4a
CCY6377	*CCY5831 + RAD61/rad61Δ::hphNT1*	b	S288c	EV4a
CCY6379	*CCY5831 + RCR2/rcr2Δ::hphNT1*	b	S288c	EV4a
CCY6382	*CCY5831 + SAC6/sac6Δ::hphNT1*	b	S288c	EV4a
CCY6383	*CCY5831 + Chr4R-497,791 bp::KanMX4::TEL, SNQ2/snq2Δ::KanMX4*	b	S288c	5b
CCY6412	*CCY5831 + KRE28/kre28Δ::hphNT1*	b	S288c	EV4a, S3
CCY6415	*CCY5831 + VPS72/vps72Δ::hphNT1*	b	S288c	EV4a, S3
CCY6418	*CCY5831 + SMT3/smt3Δ::hphNT1*	b	S288c	EV4a, S3
CCY6433	*CCY5833 + Chr5L-151,182 bp::KanMX4::TEL*	b	S288c	4c, d, e, 5a, b, 6d, S2
CCY6436	*CCY5833 + Chr5R-152,907 bp::KanMX4::TEL*	b	S288c	4c, d, e, 5a, b, 6d, S2
CCY6553	*CCY5833 + Chr5L-76,019 bp::hphNT1::TEL*	b	S288c	EV4a
CCY6556	*CCY5833 + Chr5L-133,182 bp::KanMX4::TEL*	b	S288c	EV4a
CCY6564	*CCY5833 + Chr5R-415,848 bp::hphNT1::TEL*	b	S288c	EV4a
CCY6567	*CCY5833 + Chr5R-364,508 bp::hphNT1::TEL*	b	S288c	EV4a
CCY6569	*CCY5833 + Chr5R-301,923 bp::hphNT1::TEL*	b	S288c	EV4a
CCY6572	*CCY5833 + Chr5R-242,199 bp::hphNT1::TEL*	b	S288c	EV4a
CCY6575	*CCY5833 + Chr5R-182,637 bp::hphNT1::TEL*	b	S288c	EV4a
CCY6900	*CCY5833 + GIM4/gim4Δ::KanMX4*	b	S288c	S3a, S4
CCY7088	*CCY5833 + BIM1/bim1Δ::KanMX4*	b	S288c	S3a, S4
CCY5601	*CCY5841 + TOR1/tor1Δ::KanMX4*	b	S288c	S3b
CCY5603	*MATα HIS3 LEU2, LYS2, URA3, Δtor1::KanMX4*	b	S288c	S3b
CCY5604	*MATα HIS3 LEU2, LYS2, URA3, Δtor1::KanMX4*	b	S288c	S3b
CCY6582	*CCY5841 + Chr10L-435,981 bp::KanMX4::TEL*	b	S288c	4c,d, e, S4a, b, EV4a, b

CCY6584	*CCY5841 + Chr10R-436,802 bp::KanMX4::TEL*	b	S288c	4c,d, e, S2a, b, EV4a, b
CCY6903	*CCY5841 + PFD1/pfd1Δ::KanMX4*	b	S288c	S3b
CCY7063	*CCY5841 + Chr10L-72,703 bp::hphNT1::TEL*	b	S288c	EV4a, b
CCY7066	*CCY5841 + Chr10L-220,012 bp::KanMX4::TEL*	b	S288c	EV4a, b
CCY7126	*CCY5841 + Chr10L-110,725 bp::hphNT1::TEL*	b	S288c	EV4a, b
CCY7129	*CCY5841 + Chr10L-328,956 bp::hphNT1::TEL*	b	S288c	EV4a, b
CCY6620	*CCY5845 + Chr12L-150,711 bp::KanMX4::TEL*	b	S288c	4d, e, 6d, S2, EV4a
CCY6621	*CCY5845 + Chr12R-151,725 bp::KanMX4::TEL*	b	S288c	4d, e, 6d, S2, EV4a
CCY5932	*CCY5845 + Chr12R-489,957 bp::KanMX4::TEL*	b	S288c	EV4a
CCY6634	*CCY5847 + Chr13L-267,931 bp::KanMX4::TEL*	b	S288c	4d
CCY6635	*CCY5847 + Chr13R-268,791 bp::KanMX4::TEL*	b	S288c	4d
CCY7095	*CCY5847 + TUB3/tub3::hphNT1*	b	S288c	EV3d, S3a
CCY7098	*CCY5847 + CIN4/cin4::hphNT1*	b	S288c	EV3d, S3a
CCY6587	*CCY5848 + Chr14L-627,772 bp::KanMX4::TEL*	b	S288c	4d, e, 6d, EV4a
CCY6589	*CCY5848 + Chr14R-629,032 bp::KanMX4::TEL*	b	S288c	4d, e, 6d, EV4a
CCY7054	*CCY5848 + Chr14L-220,164 bp::KanMX4::TEL*	b	S288c	EV4a
CCY7055	*CCY5848 + Chr14L-120,998 bp::KanMX4::TEL*	b	S288c	EV4a
CCY7058	*CCY5848 + Chr14L-320,127 bp::hphNT1::TEL*	b	S288c	EV4a
CCY6815	*CCY5848 + MCK1/mck1Δ::hphNT1*	b	S288c	S3a
CCY6847	*CCY5848 + MCK1/mck1Δ::hphNT1*	b	S288c	S3a
CCY7322	*MATα, ura3Δ0, pRS316; set1Δ0*	b	S288c	6c
CCY8519	*MATα, ura3Δ0, pRS316; HTB2-mNeonGreen::NAT*	b	S288c	EV 5e
CCY8521	*MATα, ura3Δ0, pRS316; HTB2-mCherry::HYG*	b	S288c	EV 5e
CCY8523	*MATα, HIS3;leu2D0; lys2D0;URA3; pGalCEN2::L*	b	S288c	EV 5e
CCY8525	*MATα, HIS3;leu2D0; lys2D0;URA3; pGalCEN4::LYS2::pCC644::LEU2; HTB2-mCherry::HYG*	b	S288c	EV 5e
CCY8527	*MATα, his3D1;leu2D0; lys2D0;ura3D2; pGalCEN4::LYS2::pCC644::LEU2 ; pGalCEN2::URA3::pCC631::HIS3; HTB2-mNeonGreen::NAT*	b	S288c	EV 5e
CCY8528	*MATα, his3D1;leu2D0; lys2D0;ura3D2; pGalCEN4::LYS2::pCC644::LEU2 ; pGalCEN2::URA3::pCC631::HIS3; HTB2-mCHerry::HYG*	b	S288c	EV 5e
CCY7434	*MATα/Mata; his3D1/HIS3; leu2D0/LEU2; lys2D0/LYS; ura3D0/URA3*	b	S288c	EV 3h, i
CCY8693	*MATalpha/ MATa; HIS3/his3D1; LEU2; LYS2; ura3D0; Δtub3/Δtub3::KANMX*	b	S288c	EV 3h, i
CCY8694	*MATα/Mata; his3D1; LEU2/leu2D0; URA3/ ura3D0; LYS2l/ys2D0;TRP1::TUB3::KanMX::TRP1/TRP1::TUB3::KanMX::TRP1*	b	S288c	EV 3h, i

CCY8695	*MATα/Mata; his3D1; LEU2/leu2D0; URA3/ ura3D0; LYS2l/ys2D0;TRP1::TUB3::KanMX::TRP1*	b	S288c	EV 3h, i
CCY8696	*MATα/Mata; his3D1; LEU2/leu2D0; URA3/ ura3D0; LYS2l/ys2D0;TRP1::TUB3::KanMX::TRP1*	b	S288c	EV 3h, i
CCY8697	*MATalpha/ MATa; HIS3/his3D1; LEU2; LYS2; ura3D0; TUB3/∆tub3::KANMX*	b	S288c	EV 3h, i

**Key**	**Source**
a	Brachmann CB et al., Yeast. (1998)
b	This study
*	Strain from the chromosome loss collection (Sup. Figure1B)
**	Strain from the chromosome gain collection (Sup. Figure1B)

Appendix Table S3. Plasmids used in this Study

Plasmid	Description	Source
pCC237	pFA6a-KanMX4	a
pCC239	pFA6a-hphNT1	b
pKA52	HIS3 integration plasmid with part of URA3 inserted at the MCS. Can insert HIS3 into a URA3 locus. Used for making disomic strains.	c
pGALCEN- JC3-13	For replacing centromeres with CEN3 under the GAL-10 promoter (URA3)	c
pCC658	For replacing centromeres with CEN3 under the GAL-10 promoter (LYS2)	d
pCC644	LEU2 integration vector with bases 3043-3538 of LYS2 for Disruption of the LYS2 gene. Used for making disomic strains.	d
pBS35	C-Terminal mCherry tag with a Hygromycin resistance	e
pCC778	C-Terminal mNeonGreen with a NrsR resistance resistance (mNeonGreen cloned from pBS34)	e, f
pCC943	For integreating TUB3 plus promoter into the TRP1 locus) (backbone was cloned from pRS304)	f

Key

a	Bähler et al., Yeast. (1998)
b	Janke *et al. Yeast* (2004).
c	Anders *et al. BMC Genet* (2009).
d	Ravichandran *et al. Genes Dev.* (2018).
e	Hailey et al. *Methods Enzymol. (2002)*
f	this study

Appendix Table S4 Primers used in this study for chromosomal deletions and qPCR

Primer	Sequence	Description	Cut site [bp]	Source
Kan_overlap-FP	**GGCCGCCAGCTGAAGCTTCGTACGCTGCAG**CCTTGACAGTCTTGACGTGC	Universal forward primer for pFA6a-KanMX4 amplification	-	a
Kan_overlap-RP	**CCCCAACCCCAACCCCAACCCCAACCCCAACCCCAA**CGCACTTAACTTCGCATCTG	Universal reverse primer - telomeric seed sequence	-	a
C4S1_del-FP	CCACGAATATCCATCGTTGTTT	Amplifying homology region on chromosome 4 right arm	Chr4: 107189	a
C4S1_del-RP	**CTGCAGCGTACGAAGCTTCAGCTGGCGGCC**AGCCCACGATGAACTCTAAACT	Amplifying homology region on chromosome 4 right arm	Chr4: 1071899	a
C4S3_del-FP	TGGAAAATGCTGGCAAAGTGT	Amplifying homology region on chromosome 4 right arm	Chr4: 117236	a
C4S3_del-RP	**CTGCAGCGTACGAAGCTTCAGCTG**TCTCCTTGACCAATGCACGT	Amplifying homology region on chromosome 4 right arm	Chr4: 1172361	a
OCC3609	ACGAGTGTGTAATTACCC	Amplifying homology region on chromosome 4 right arm	Chr4: 138493	b
OCC3610	**CTGCAGCGTACGAAGCTTCAGCTG**AACAAAAGCAGGTACAGG	Amplifying homology region on chromosome 4 right arm	Chr4: 1384937	b
OCC3390	AGTGATTTCCACACTTTGG	Amplifying homology region on chromosome 4 right arm	Chr4: 899451	b
OCC3391	**CTGCAGCGTACGAAGCTTCAGCTG**GCCAGGAGTGGTTAATGT	Amplifying homology region on chromosome 4 right arm	Chr4: 899451	b
OCC3392	GCAATGGCACAATATGGA	Amplifying homology region on chromosome 4 right arm	Chr4: 700500	b
OCC3393	**CTGCAGCGTACGAAGCTTCAGCTG**CAATGGATCGAACGTGAG	Amplifying homology region on chromosome 4 right arm	Chr4: 700500	b
OCC3436	CGTGTTCTCTTCTCATCG	Amplifying homology region on chromosome 4 right arm	Chr4: 598898	b
OCC3437	**CTGCAGCGTACGAAGCTTCAGCTG**TGATCGCAGACTAATTGAG	Amplifying homology region on chromosome 4 right arm	Chr4: 598898	b
OCC3394	TCGCTTATTGACATTGGC	Amplifying homology region on chromosome 4 right arm	Chr4: 497791	b
OCC3395	**CTGCAGCGTACGAAGCTTCAGCTG**AGGTAGTGTGGATCAGTG	Amplifying homology region on chromosome 4 right arm	Chr4: 497791	b
OCC3396	AAAGCACTACCTAGGAGC	Amplifying homology region on chromosome 4 right arm-full	Chr4: 449859	b
OCC3397	**CTGCAGCGTACGAAGCTTCAGCTG**CAGGTACAGTCCTCTAGG	Amplifying homology region on chromosome 4 right arm-full	Chr4: 449859	b
OCC3398	**GTCGACCTGCAGCGTACGAAGCTTCAGCTG**ATAGTGGTTGACATGCTG	Amplifying homology region on chromosome 4 left arm-full	Chr4: 449243	b

OCC3399	GTTACTATTTCTGGCTCGTG	Amplifying homology region on chromosome 4 left arm-full	Chr4: 449243	b
OCC3454	**CTGCAGCGTACGAAGCTTCAG** **CTG**ATAGGGACCATACACACG	Amplifying homology region on chromosome 2 left arm-full	Chr2: 237423	b
OCC3455	CAGTCTGTTTGAACGTTC	Amplifying homology region on chromosome 2 left arm-full	Chr2: 237423	b
OCC3456	CTTCCTATGCACTAGACC	Amplifying homology region on chromosome 2 right arm-full	Chr2: 238979	b
OCC3457	**CTGCAGCGTACGAAGCTTCAG** **CTG**AGTTTGAAACCCTCTGAG	Amplifying homology region on chromosome 2 right arm-full	Chr2: 238979	b
OCC3853	ATTCCTTGCAAACATGGA	Amplifying homology region on chromosome 2 right arm	Chr2: 713057	b
OCC3854	**CTGCAGCGTACGAAGCTTCAG** **CTG**AGTTTGTTGATCCACAGG	Amplifying homology region on chromosome 2 right arm	Chr2: 713057	b
OCC3800	AACGTTGCGAATAAAACG	Amplifying homology region on chromosome 2 right arm	Chr2: 686861	b
OCC3801	**CTGCAGCGTACGAAGCTTCAG** **CTG**TGGCAAAAACACTGTCTC	Amplifying homology region on chromosome 2 right arm	Chr2: 686861	b
OCC3851	CTCTTTCGCTTAGGTTCC	Amplifying homology region on chromosome 2 right arm	Chr2: 665194	b
OCC3852	**CTGCAGCGTACGAAGCTTCAG** **CTG**ACCATCGTTAAAGCTGTC	Amplifying homology region on chromosome 2 right arm	Chr2: 665194	b
OCC3706	CACCGTAGAGAGGTAGAG	Amplifying homology region on chromosome 2 right arm	Chr2: 642519	b
OCC3707	**CTGCAGCGTACGAAGCTTCAG** **CTG**TGTTTTGCTTTTTCCTGC	Amplifying homology region on chromosome 2 right arm	Chr2: 642519	b
OCC3802	TGTTCGGATGTACTTTGC	Amplifying homology region on chromosome 2 right arm	Chr2: 589726	b
OCC3803	**CTGCAGCGTACGAAGCTTCAG** **CTG**TCCCAGCTCTTGAAACTC	Amplifying homology region on chromosome 2 right arm	Chr2: 589726	b
OCC3708	GAAAATCATGCGAAGGAG	Amplifying homology region on chromosome 2 right arm	Chr2: 537847	b
OCC3709	**CTGCAGCGTACGAAGCTTCAG** **CTG**ATGTTAATACACCGGTGC	Amplifying homology region on chromosome 2 right arm	Chr2: 537847	b
OCC3710	ATATTCCAGTTGCGAACG	Amplifying homology region on chromosome 2 right arm	Chr2: 436902	b
OCC3711	**CTGCAGCGTACGAAGCTTCAG** **CTG**CCAATTCAACTTGTCTGC	Amplifying homology region on chromosome 2 right arm	Chr2: 436902	b
OCC3712	GAGGGTACTCAATTGACG	Amplifying homology region on chromosome 2 right arm	Chr2: 341042	b
OCC3713	**CTGCAGCGTACGAAGCTTCAG** **CTG**AACTGAGAAGCTGAATGG	Amplifying homology region on chromosome 2 right arm	Chr2: 341042	b

OCC3400	**GTCGACCTGCAGCGTACGAAG CTTCAGCTG**CATCGTGTAGTCA AGCAG	Amplifying homology region on chromosome 5 left arm-full	Chr5: 151182	b
OCC3401	CTCGCCATTCAATGACAG	Amplifying homology region on chromosome 5 left arm-full	Chr5: 151182	b
OCC3402	AAGCCGCACATAAATAGC	Amplifying homology region on chromosome 5 right arm-full	Chr5: 152907	b
OCC3403	**CTGCAGCGTACGAAGCTTCAG CTG**GTTCCTATCTCAGGGAGAG	Amplifying homology region on chromosome 5 right arm-full	Chr5: 152907	b
OCC3446	**CTGCAGCGTACGAAGCTTCAG CTG**TTTAGGTGCGTCATTACC	Amplifying homology region on chromosome 5 left arm	Chr5: 76019	b
OCC3447	CCATAGTATCCATCTCAGC	Amplifying homology region on chromosome 5 left arm	Chr5: 76019	b
OCC3444	**CTGCAGCGTACGAAGCTTCAG CTG**GGCATACTATACCACACG	Amplifying homology region on chromosome 5 left arm	Chr5: 133182	b
OCC3445	AAACGGTTTCAACCATGG	Amplifying homology region on chromosome 5 left arm	Chr5: 133182	b
OCC3742	GAAGGAACGTAGAGTCAC	Amplifying homology region on chromosome 5 right arm	Chr5: 415848	b
OCC3743	**CTGCAGCGTACGAAGCTTCAG CTG**CTATATCGTGGCACTTCTG	Amplifying homology region on chromosome 5 right arm	Chr5: 415848	b
OCC3442	TATCTCTTCCAGACCAGG	Amplifying homology region on chromosome 5 right arm	Chr5: 364508	b
OCC3443	**CTGCAGCGTACGAAGCTTCAG CTG**AGAAGAGATAGGCCTCTG	Amplifying homology region on chromosome 5 right arm	Chr5: 364508	b
OCC3613	GTGTTCGGGTTAATGAGG	Amplifying homology region on chromosome 5 right arm	Chr5: 301923	b
OCC3614	**CTGCAGCGTACGAAGCTTCAG CTG**CACATAAAGTTGCGTTCC	Amplifying homology region on chromosome 5 right arm	Chr5: 301923	b
OCC3611	CTTAGTCGTTACCTGGTG	Amplifying homology region on chromosome 5 right arm	Chr5: 242199	b
OCC3612	**CTGCAGCGTACGAAGCTTCAG CTG**ACTACCGAAATCGAAGCA	Amplifying homology region on chromosome 5 right arm	Chr5: 242199	b
OCC4085	CATGTCGCAGGTAACATC	Amplifying homology region on chromosome 5 right arm	Chr5: 182637	b
OCC4086	**CTGCAGCGTACGAAGCTTCAG CTG**TTTGCTCTCGGTTTTAGC	Amplifying homology region on chromosome 5 right arm	Chr5: 182637	b
OCC3404	**GTCGACCTGCAGCGTACGAAG CTTCAGCTG**ATGCCTCTGGATC TACAC	Amplifying homology region on chromosome 10 left arm-full	Chr10: 435981	b
OCC3405	GATACGTAGATACAGTCACC	Amplifying homology region on chromosome 10 left arm-full	Chr10: 435981	b
OCC3406	GAGTTGTTGAGAACGAGT	Amplifying homology region on chromosome 10 right arm-full	Chr10: 436802	b

OCC3407	**CTGCAGCGTACGAAGCTTCAGCTG**TTTGCGTCTCGGAATCAG	Amplifying homology region on chromosome 10 right arm-full	Chr10: 436802	b
OCC3448	**CTGCAGCGTACGAAGCTTCAGCTG**GTCACTCTACTTTGAGGC	Amplifying homology region on chromosome 10 left arm	Chr10: 72703	b
OCC3449	TCGCTTAGACTTAGCAGC	Amplifying homology region on chromosome 10 left arm	Chr10: 72703	b
OCC3485	AGGTAGCACAGAAAGTAGTG	Amplifying homology region on chromosome 10 left arm	Chr10: 110725	b
OCC3486	**CTGCAGCGTACGAAGCTTCAGCTG**GGATGTGTTACGATAGAAAGC	Amplifying homology region on chromosome 10 left arm	Chr10: 110725	b
OCC3450	**CTGCAGCGTACGAAGCTTCAGCTG**CCATTGTATAGCCTATCGG	Amplifying homology region on chromosome 10 left arm	Chr10: 220012	b
OCC3451	ACGTCTGTATCACGTTCC	Amplifying homology region on chromosome 10 left arm	Chr10: 220012	b
OCC3489	CATACGGGAAGTGAAAGC	Amplifying homology region on chromosome 10 left arm	Chr10: 328956	b
OCC3490	**CTGCAGCGTACGAAGCTTCAGCTG**CGCCAATAACAATTGATGG	Amplifying homology region on chromosome 10 left arm	Chr10: 328956	b
OCC3420	**GTCGACCTGCAGCGTACGAAGCTTCAGCTG**AAATGTGCTCTCCTCTCC	Amplifying homology region on chromosome 12 left arm-full	Chr12: 150711	b
OCC3421	TAGCATACACCAGTTTGG	Amplifying homology region on chromosome 12 left arm-full	Chr12: 150711	b
OCC3422	ATGAGGTACTGACACTGC	Amplifying homology region on chromosome 12 right arm-full	Chr12: 151725	b
OCC3423	**CTGCAGCGTACGAAGCTTCAGCTG**CGAGAAATACCATCTGCG	Amplifying homology region on chromosome 12 right arm-full	Chr12: 151725	b
OCC3424	GACGCCTTATTCGTATCC	Amplifying homology region on chromosome 12 right arm	Chr12: 489957	b
OCC3425	**CTGCAGCGTACGAAGCTTCAGCTG**GGTAGATAGGACAGATGC	Amplifying homology region on chromosome 12 right arm	Chr12: 489957	b
OCC3315	**GTCGACCTGCAGCGTACGAAGCTTCAGCTG**TGCAATCTGTCTATGAACC	Amplifying homology region on chromosome 13 left arm-full	Chr13: 267931	b
OCC3317	TGAGCGGTTCTAGAGCTA	Amplifying homology region on chromosome 13 left arm-full	Chr13: 267931	b
OCC3318	**GTCGACCTGCAGCGTACGAAGCTTCAGCTG**TGAGCGGTTCTAGAGCTA	Amplifying homology region on chromosome 13 right arm-full	Chr13: 268791	b
OCC3319	TTCTTGTAGCTGTTGAGC	Amplifying homology region on chromosome 13 right arm-full	Chr13: 268791	b
OCC3408	**GTCGACCTGCAGCGTACGAAGCTTCAGCTG**GCTTTACCCTTGACTACG	Amplifying homology region on chromosome 14 left arm-full	Chr14: 627772	b

OCC3409	CCTCCATTGCTCTCTACG	Amplifying homology region on chromosome 14 left arm- full	Chr14: 627772	b
OCC3410	GGTTTATGCTACCCTGCT	Amplifying homology region on chromosome 14 right arm- full	Chr14: 629032	b
OCC3411	CTGCAGCGTACGAAGCTTCAGCTGTTGATTCAGGGTATGCGA	Amplifying homology region on chromosome 14 right arm- full	Chr14: 629032	b
OCC3615	CTGCAGCGTACGAAGCTTCAGCTGAACGCAGTTAGGATGTAC	Amplifying homology region on chromosome 14 left arm	Chr14: 120998	b
OCC3616	TCATAACAGACACCAGCT	Amplifying homology region on chromosome 14 left arm	Chr14: 120998	b
OCC3617	CTGCAGCGTACGAAGCTTCAGCTGAGAGATTCGACACTTGGT	Amplifying homology region on chromosome 14 left arm	Chr14: 220164	b
OCC3618	CATAGAACGCCGAAACAG	Amplifying homology region on chromosome 14 left arm	Chr14: 220164	b
OCC3452	CTGCAGCGTACGAAGCTTCAGCTGACATCATCTTTTTGTGCTG	Amplifying homology region on chromosome 14 left arm	Chr14: 320127	b
OCC3453	TGATTCACAAATGCCTGG	Amplifying homology region on chromosome 14 left arm	Chr14: 320127	b
OCC919	ACAGCTTCTAAACGTTCCGTGTGC	ChrI Left arm (Forward) - qPCR		c
OCC920	GCGGTGTGTGGATGATGGTTTCAT	ChrI Left arm (Reverse) - qPCR		c
OCC921	GCACTTGATCCATGTAGCCATACTCG	ChrI Right arm (Forward) - qPCR		c
OCC922	TTCGGGTGACCCTTATGGCATTCT	ChrI Right arm (Reverse) - qPCR		c
OCC923	TTTCAGGATCACGAGCGCCATCTA	ChrII Left arm (Forward) - qPCR		c
OCC924	CGGCAAGTGTCTCACTGTTGCATT	ChrII Left arm (Reverse) - qPCR		c
OCC1008	TGGCTAAACATGCAGCCACACATA	ChrII Right arm (Forward) - qPCR		b
OCC1009	TTCAAAATACCCAACGGGCAGCTG	ChrII Right arm (Reverse) - qPCR		b
OCC927	TTGTTTCTGTCCTTGCCACAGCTC	ChrIII Left arm (Forward) - qPCR		c
OCC928	AGCGCCTTTACCTCAACCTACCAT	ChrIII Left arm (Reverse) - qPCR		c
OCC929	ATCCAGCCCGCACAAATGAATACC	ChrIII Right arm (Forward) - qPCR		c
OCC930	AGAATGGAACACTCCTCACCACGA	ChrIII Right arm (Reverse) - qPCR		c
OCC931	AGCCCTAGTTGCAGATCATCGTGT	ChrIV Left arm (Forward) - qPCR		c
OCC932	AGAATATACGGCAACAGTGCCCGA	ChrIV Left arm (Reverse) - qPCR		c
OCC933	GGCCAACAAATCTTGTACCTCGCT	ChrIV Right arm (Forward) - qPCR		c
OCC934	GTTACCGAAGAAGGCCACCAATCT	ChrIV Right arm (Reverse) - qPCR		c

OCC935	TCCGCCGGCAACTGTAACTGTAAA	ChrV Left arm (Forward) - qPCR	c
OCC936	ATAGTAACCAACGAGAGCGCGCAA	ChrV Left arm (Reverse) - qPCR	c
OCC1465	CAAGCCACTGTTGGCGTTTCAACT	ChrV Right arm (Forward) - qPCR	c
OCC1466	TTTATGTGCGGCTTTGTCAGCAGG	ChrV Right arm (Reverse) - qPCR	c
OCC937	GCGCTTATGTAAGGTTCCTGTATGGT	ChrVI Left arm (Forward) - qPCR	c
OCC938	AGTGCGGATTCATTTCCAAGCAGC	ChrVI Left arm (Reverse) - qPCR	c
OCC939	TTAACCTTGGCGTTTCAGCATCCG	ChrVI Right arm (Forward) - qPCR	c
OCC940	TGATCTTCCGCCGATTGGTGTTCA	ChrVI Right arm (Reverse) - qPCR	c
OCC941	TGTGCGTCTTCCCTAAAGCAGCTA	ChrVII Left arm (Forward) - qPCR	c
OCC942	GCATTGGATGCGATGAGATGGCAA	ChrVII Left arm (Reverse) - qPCR	c
OCC943	TTACGAGCCTTTCAGACCTGCGTA	ChrVII Right arm (Forward) - qPCR	c
OCC944	GTGAAATACGGCCGCTAAGCATCT	ChrVII Right arm (Reverse) - qPCR	c
OCC1002	AATGGGAGTGATCCGCTCAGTTCT	ChrVIII Left arm (Forward) - qPCR	b
OCC1003	GAATCTCTGCAGCAAGAGCGTAGG	ChrVIII Left arm (Reverse) - qPCR	b
OCC1004	TCATTGCAATAACAGAAAGGCCGG	ChrVIII Right arm (Forward) - qPCR	b
OCC1005	GGGAAAAGTCCGCCGGAGATAATT	ChrVIII Right arm (Reverse) - qPCR	b
OCC949	AAAGTTGGCGCTGGGTACTTTGAG	ChrIX Left arm (Forward) - qPCR	c
OCC950	AGAACTGATGGCATTTGATGGCCG	ChrIX Left arm (Reverse) - qPCR	c
OCC1469	TCTGTAGCAGAAAGAGTCTCCCGA	ChrIX Right arm (Forward) - qPCR	b
OCC1470	GGTACTCTGTGGTTTGCCCTTTGT	ChrIX Right arm (Reverse) - qPCR	b
OCC951	ATTTACCGGTTAGTGTCAGCGCCA	ChrX Left arm (Forward) - qPCR	c
OCC952	CGACAGAGTAGTTTATGCCGAGGGTT	ChrX Left arm (Reverse) - qPCR	c
OCC953	AGGCGAGTACCCTTAGCATTTCCT	ChrX Right arm (Forward) - qPCR	c
OCC954	ACGAGGCAAGTGTAGGTCCTTTGT	ChrX Right arm (Reverse) - qPCR	c
OCC955	AGCTGGTGATGAGCCAAATGTCGT	ChrXI Left arm (Forward) - qPCR	c
OCC956	TTTAGAGCAAGCGCCTTTGTGAGC	ChrXI Left arm (Reverse) - qPCR	c
OCC1471	TAGGCTTCCGGAACCACACAAGAT	ChrXI Right arm (Forward) - qPCR	c
OCC1472	AGAGGCAGCTTCCCTTCTGATTCT	ChrXI Right arm (Reverse) - qPCR	c

OCC957	TGGAGATGAAGGGTTGTCGTTGGT	ChrXII Left arm (Forward) - qPCR	c
OCC958	ACGTGTAGCGTTTCTGCTGGTCTT	ChrXII Left arm (Reverse) - qPCR	c
OCC1473	ATGGCAGGCAGGTGAATGAGATGA	ChrXII Right arm (Forward) - qPCR	c
OCC1474	AGAGTAGACCATGGGACGTCGTTT	ChrXII Right arm (Reverse) - qPCR	c
OCC1996	ATGAGTATTGAGTTCGCGGATTCG	ChrXIII Left arm (Forward) - qPCR	b
OCC1997	GTCATACTGATCCTGTGTCAGGCAC	ChrXIII Left arm (Reverse) - qPCR	b
OCC1998	CTAGCTTCAACATCGGTAGCTGGT	ChrXIII Right arm (Forward) - qPCR	b
OCC1999	TCGTTTTCCCAAGAGACGGTAAGG	ChrXIII Right arm (Reverse) - qPCR	b
OCC961	GGGATTAACAATACGGTAAAGGGACG	ChrXIV Left arm (Forward) - qPCR	c
OCC962	CAACCACTGTCAGCACAAACTCCT	ChrXIV Left arm (Reverse) - qPCR	c
OCC963	TCGCTCAGAACATCAGCGAGAGTT	ChrXIV Right arm (Forward) - qPCR	c
OCC964	GTTTCTGCGAAGGCCCTTTGTTCT	ChrXIV Right arm (Reverse) - qPCR	c
OCC967	AAGAGCCTTGAACTTCTCGGGTGA	ChrXVI Left arm (Forward) - qPCR	c
OCC968	TGATGTTCCTCTCGTTTGGCACTC	ChrXVI Left arm (Reverse) - qPCR	c
OCC1479	ACATGTGGAGCATAGCAGGCTCTT	ChrXVI Right arm (Forward) - qPCR	c
OCC1480	ATTACCTCTTTCCCACAACCGGCA	ChrXVI Right arm (Reverse) - qPCR	c
OCC3527	CGGGTGACCTGCTCCATATGC	qPCR primer for tip of ChrXIVL	b
OCC3528	AGCAACACCTTTTGATCCGATCACG	qPCR primer for tip of ChrXIVL	b
OCC3529	GCTGAGCTTACCACCTGGGTACTG	qPCR primer for tip of ChrXL	b
OCC3530	CCAGGTAATATTCCACCAAGCACACC	qPCR primer for tip of ChrXL	b
OCC3531	CCTGCGAATCTGTGATGCAAGAACG	qPCR primer for tip of ChrXIIL	b
OCC3532	GCTGTGGATGTTAGAAAACGGCTAGG	qPCR primer for tip of ChrXIIL	b
OCC3533	TGATGGAAGCGACCCAGAACTCG	qPCR primer for tip of ChrVL	b
OCC3534	TGCCCTGGAACTTAGTGTAGTTGGC	qPCR primer for tip of ChrVL	b
OCC3535	ATGGACCTTGAGGCTTCAAATCACG	qPCR primer for tip of ChrVR	b
OCC3536	AGATGGCCAGGAGGTCTTTCTGC	qPCR primer for tip of ChrVR	b
OCC3714	TGGTTTTTGGAGCGCTAGTTCTGG	qPCR primer for tip of ChrIIR	b
OCC3715	GGCCATGGACAGGGATTTGGC	qPCR primer for tip of ChrIIR	b

OCC3738	CCTCCAAGAAACAGGCATCGTCAC	qPCR primer for tip of ChrIV-R	b
OCC3739	GGCATTGTTACGCTCATACCGCT	qPCR primer for tip of ChrIV-R	b
OCC4670	ACCCATATAAATAATAATATTAATTATAACCAAAGGAAGTGATTTCATTA**ATCGATGAATTCGAGCTCC**	HTB1 S2 primer	b
OCC4671	TCTCTGAAGGTACTAGAGCTGTTACCAAGTACTCTTCCTCTACTCAAGCA**GGTCGACGGATCCCCGGG**	HTB1 S3-like YRC	b
OCC723	AAAAAATGCCACTAATAAAAAGAAAACATGACTAAATCACAATACCTAGTGAGTGACTTA**ATCGATGAATTCGAGCTCG**	HTB2 S2 primer	b
OCC724	AAACATGCCGTCTCCGAAGGTACTAGGGCTGTTACCAAATACTCCTCCTCTACTCAAGCC**GGTCGACGGATCCCCGGG**	HTB2 S3-like YRC	b

Blue indicates the telomeric seed sequence and **violet** indicates the homology between the resistance fragment and homology fragment.

**Key**	**Source**
a	Linder et al., *G3: Genes, Genomes Genet.* (2017)
b	This study
c	Pavelka et al., *Nature.* (2010)
