# Supplementary figures and images for: Combinatorial effects of multiple genes contribute to beneficial aneuploidy phenotypes
Source: EMBO Rep. 2026 Apr 11;27(10):2772–97. doi: 10.1038/s44319-026-00767-8 (PMC13219433; doi:10.1038/s44319-026-00767-8)

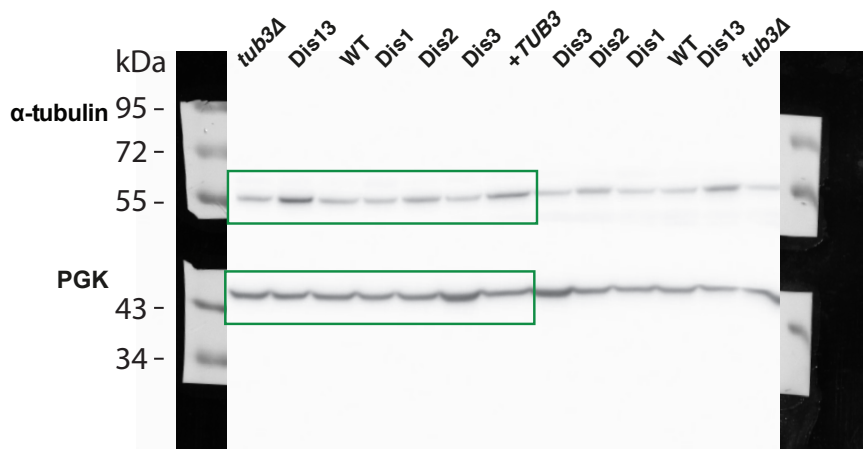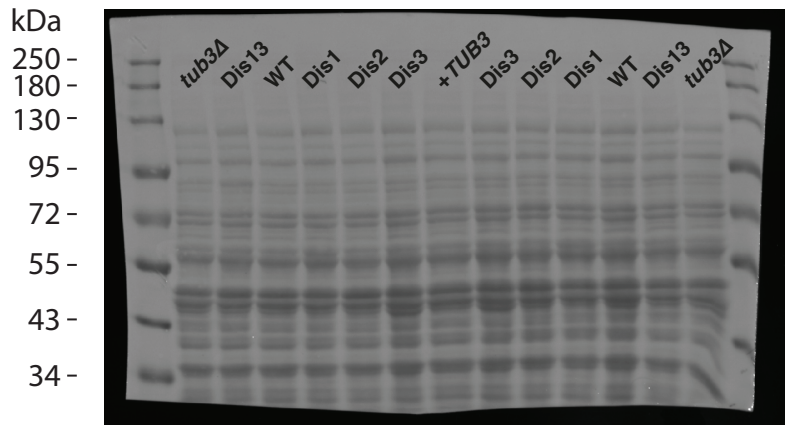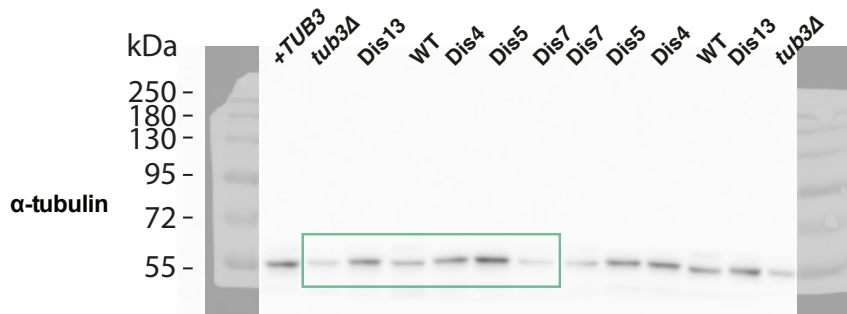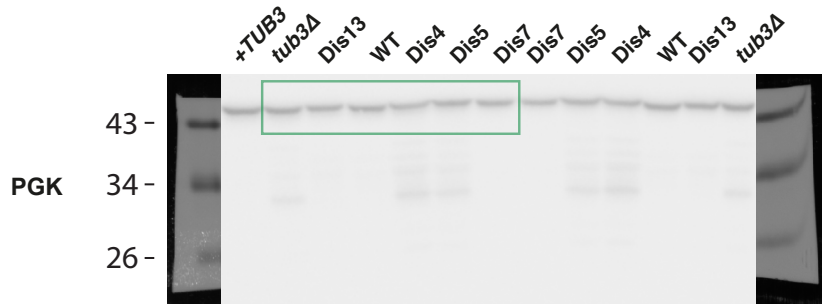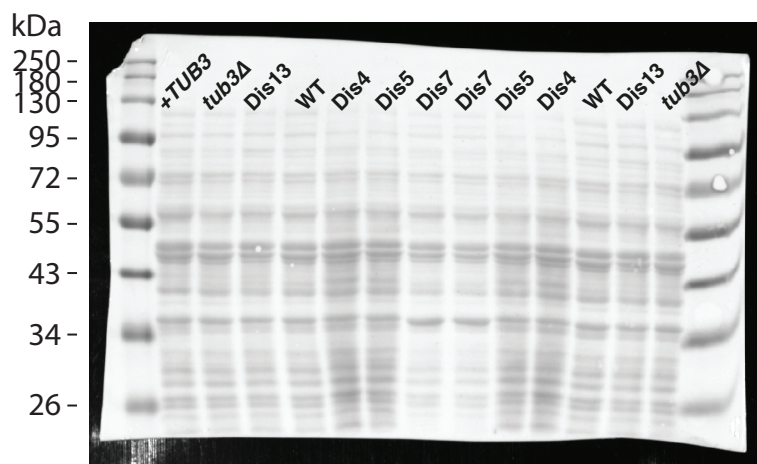

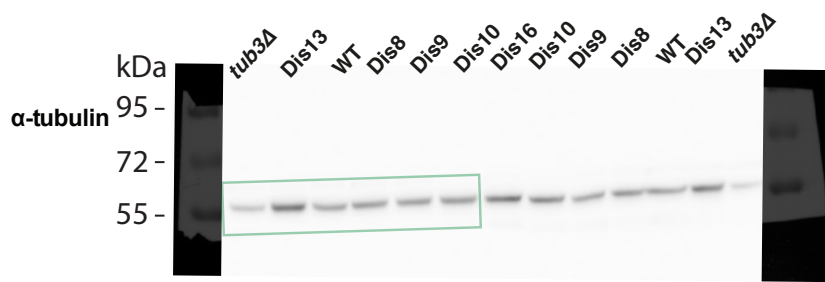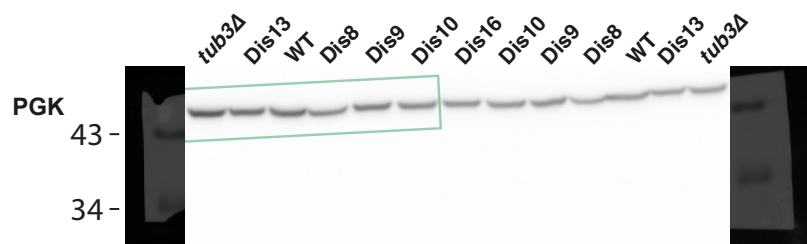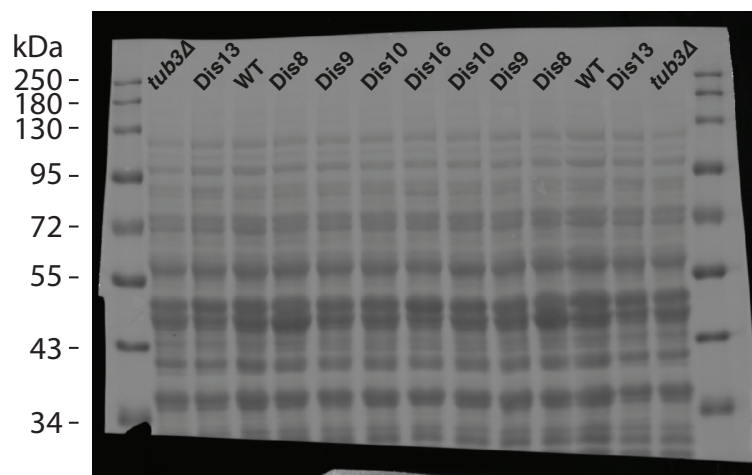

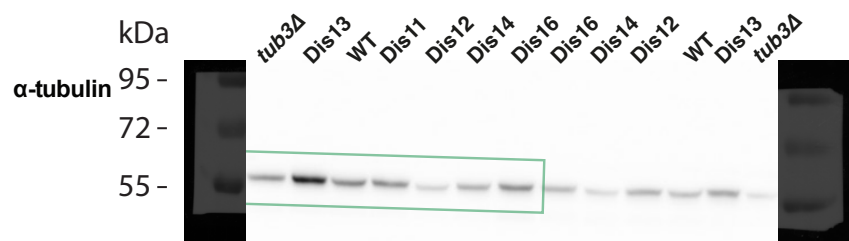

campbell 2022-11-30 11h37m15s(Chemiluminescence).raw16

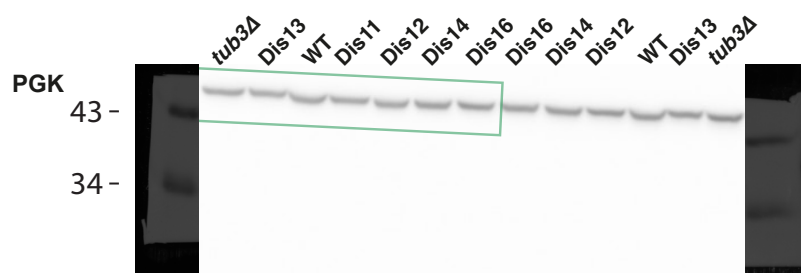

campbell 2022-11-30 11h36m33s(Chemiluminescence).raw16

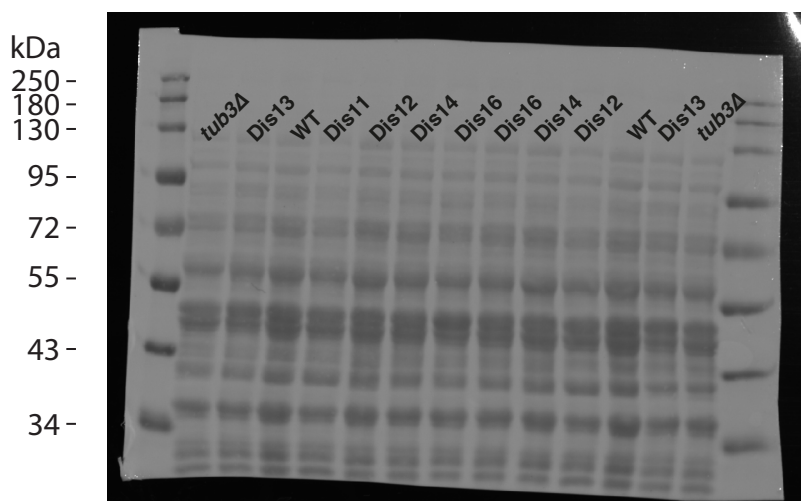

Supplement: Supplementary file 6 — Source data Fig. 3 [file 44319_2026_767_MOESM6_ESM.zip › Figure 3/3B/Figure 3B image data- blot.pdf]

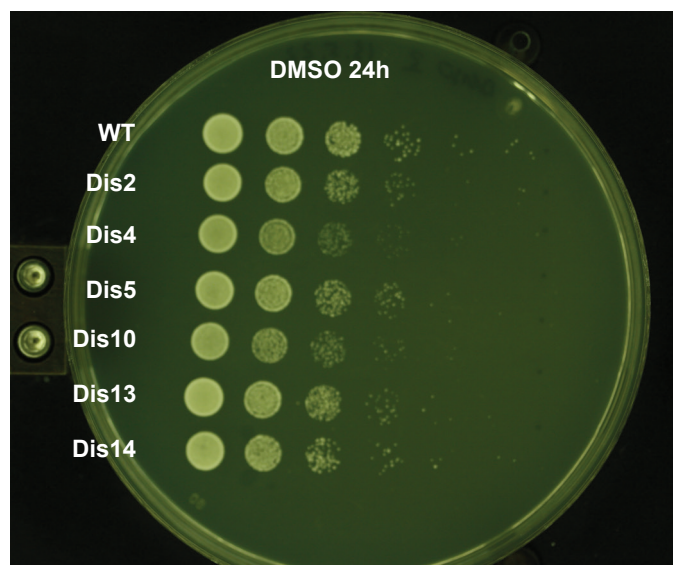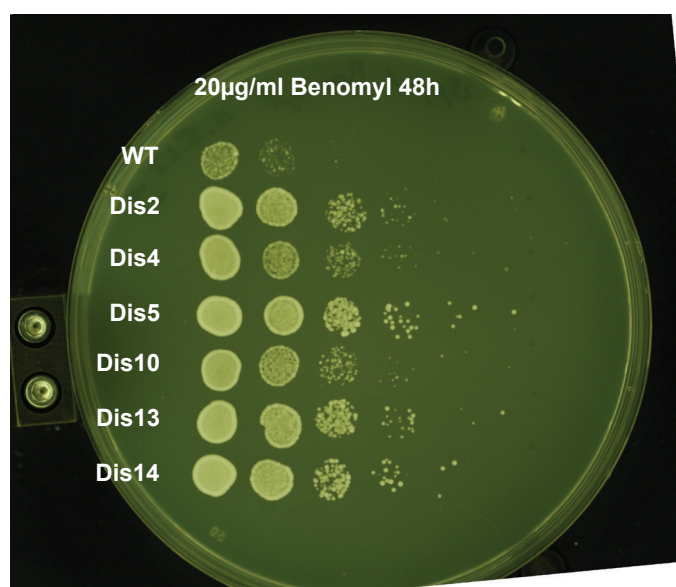

Supplement: Supplementary file 6 — Source data Fig. 3 [file 44319_2026_767_MOESM6_ESM.zip › Figure 3/3A/Figure 3A macroscopic photos.pdf]

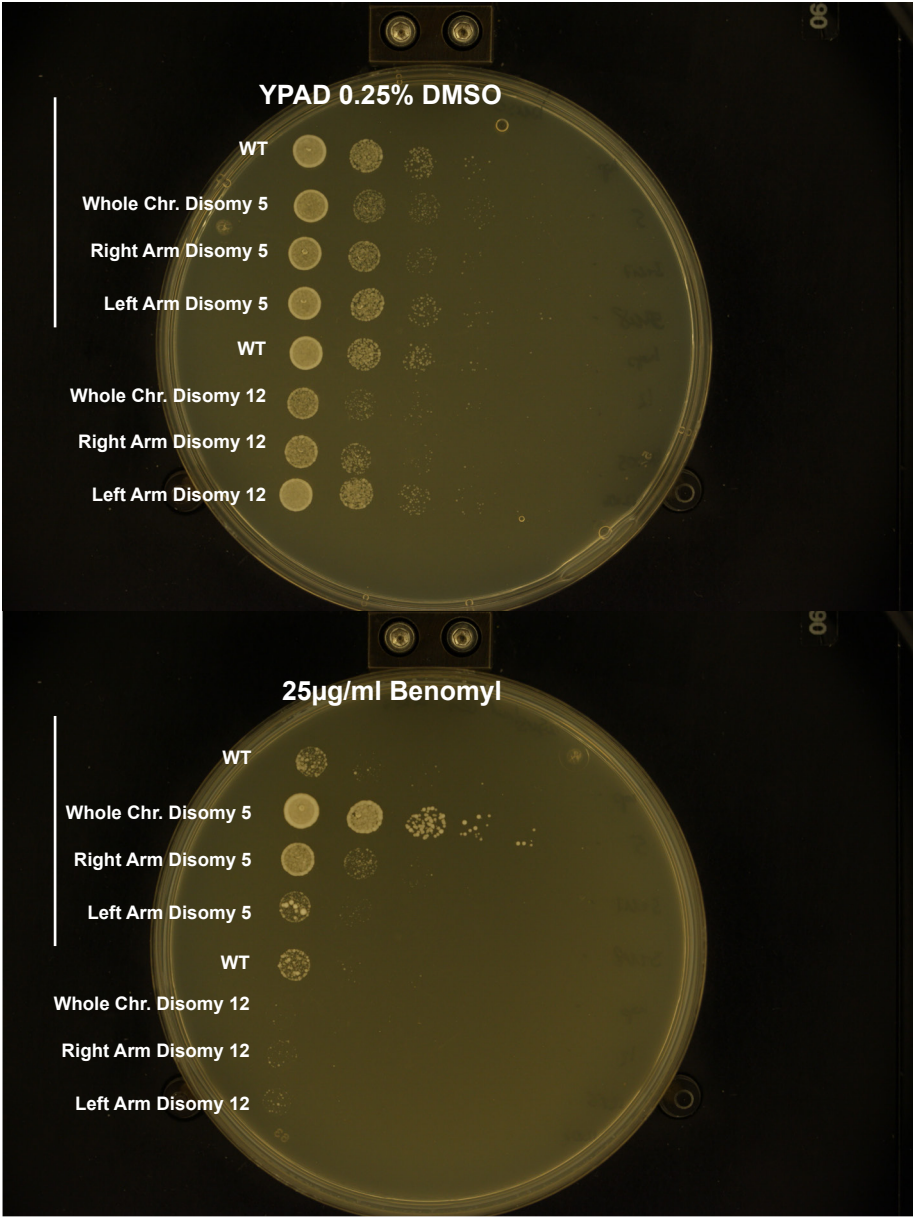

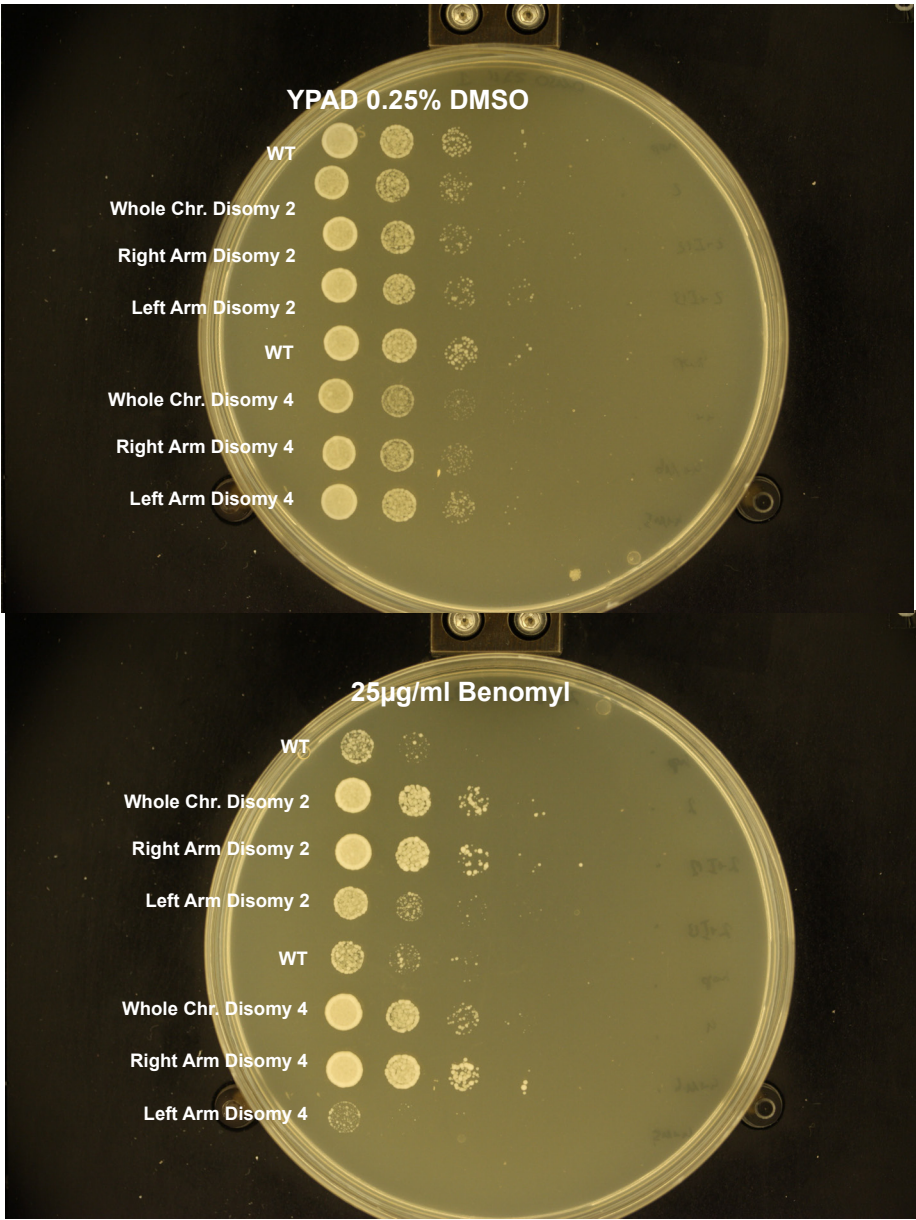

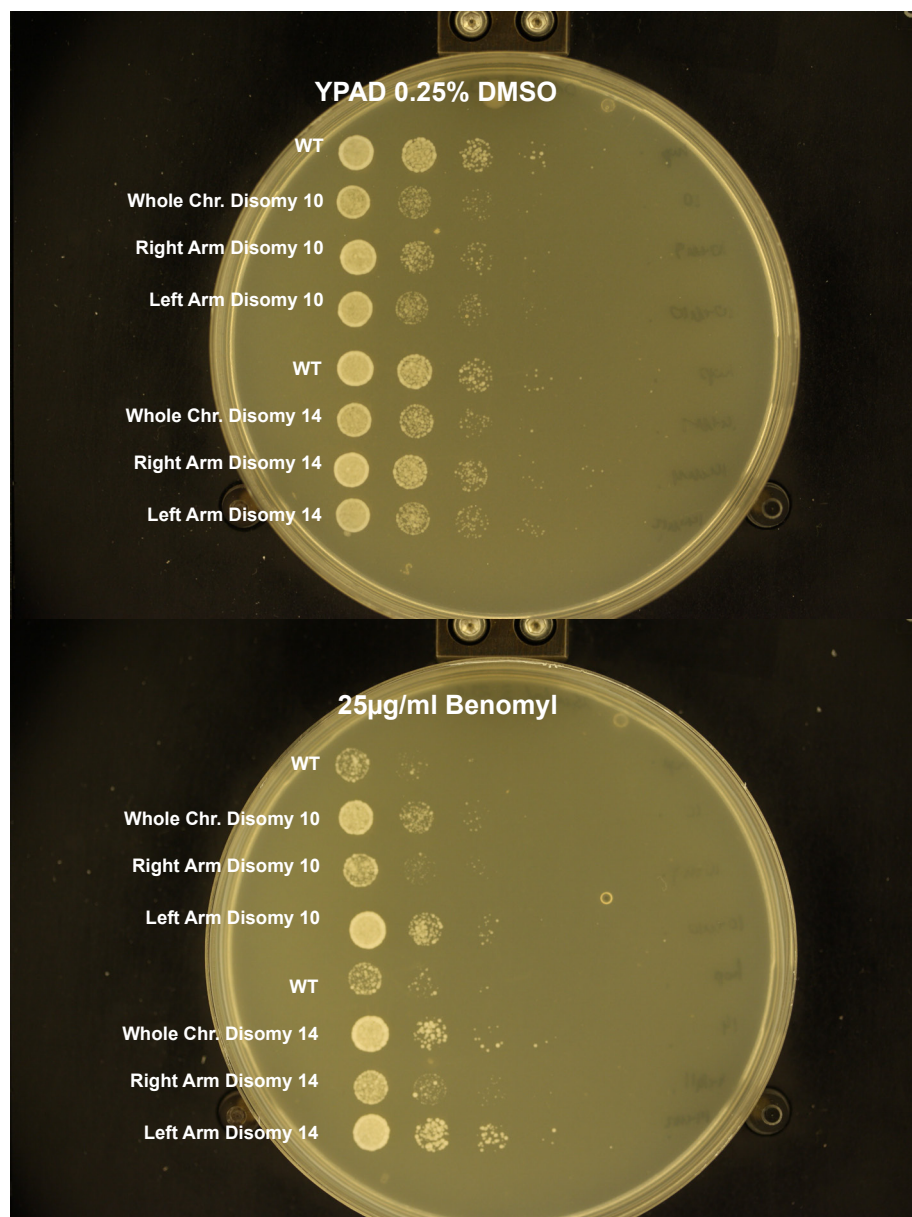

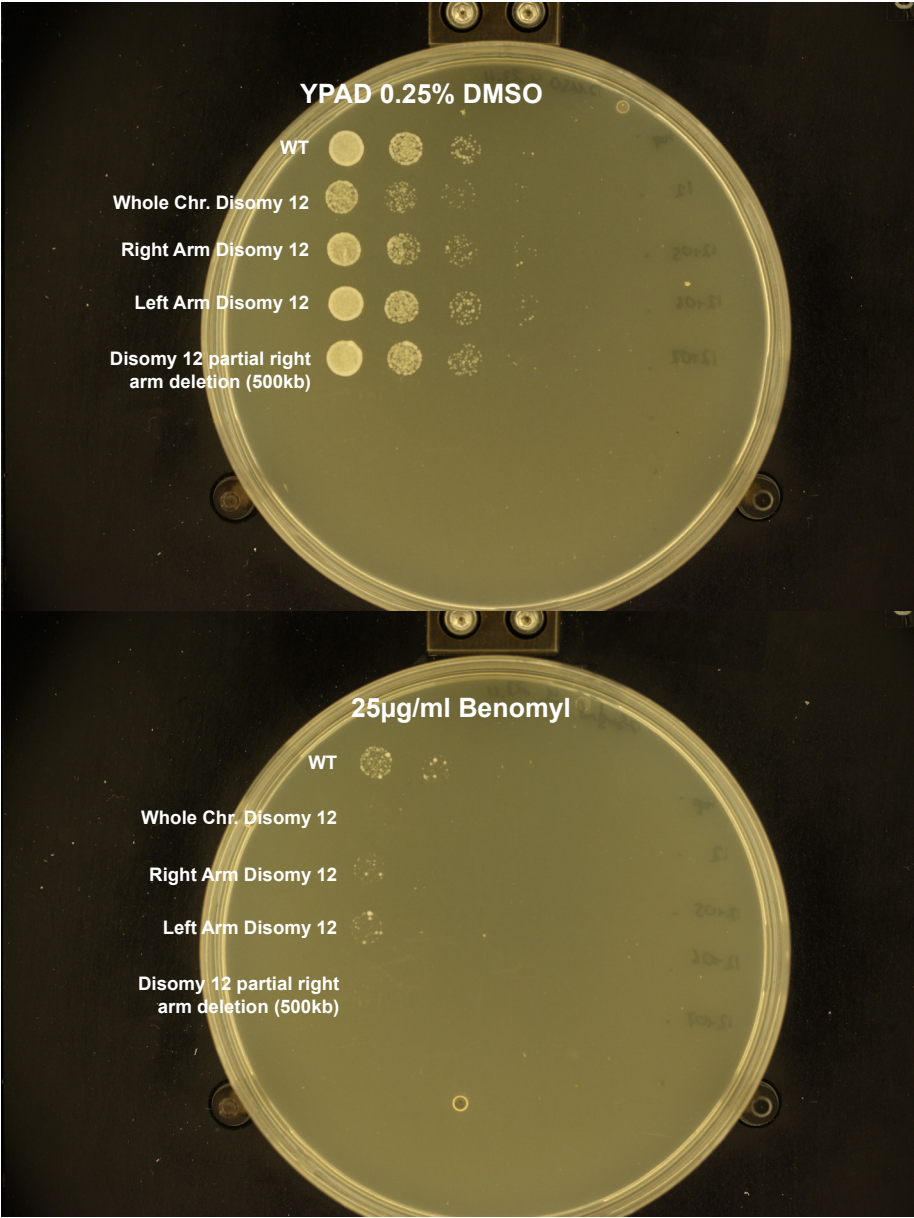

Supplement: Supplementary file 7 — Source data Fig. 4 [file 44319_2026_767_MOESM7_ESM.zip › Figure 4/4D/4D macroscopic photos.pdf]

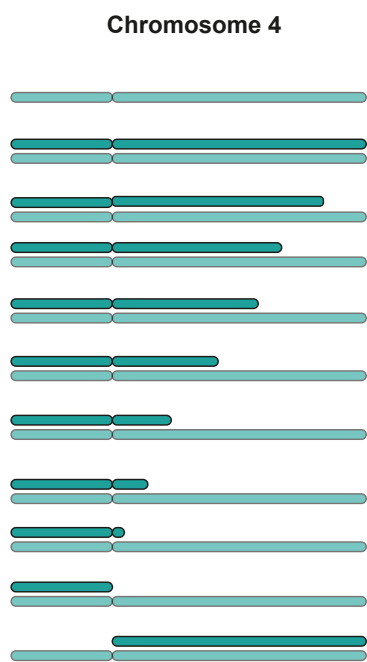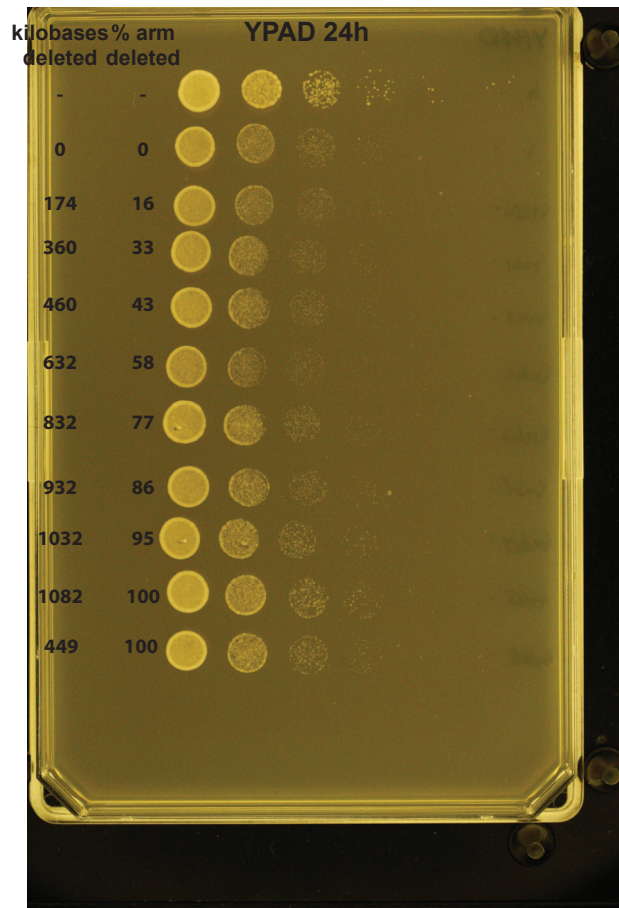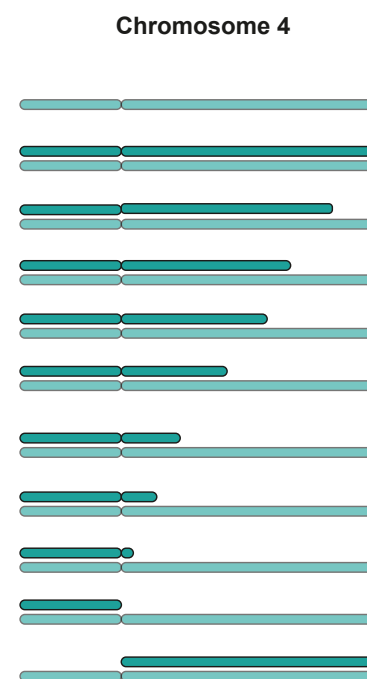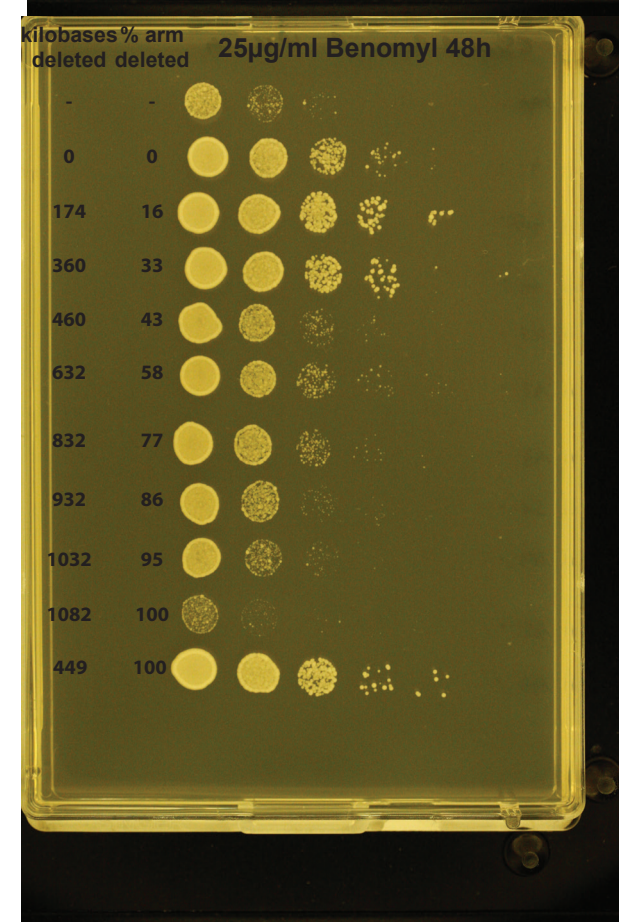

Supplement: Supplementary file 8 — Source data Fig. 5 [file 44319_2026_767_MOESM8_ESM.zip › Figure 5/5A/5A macroscopic photos.pdf]

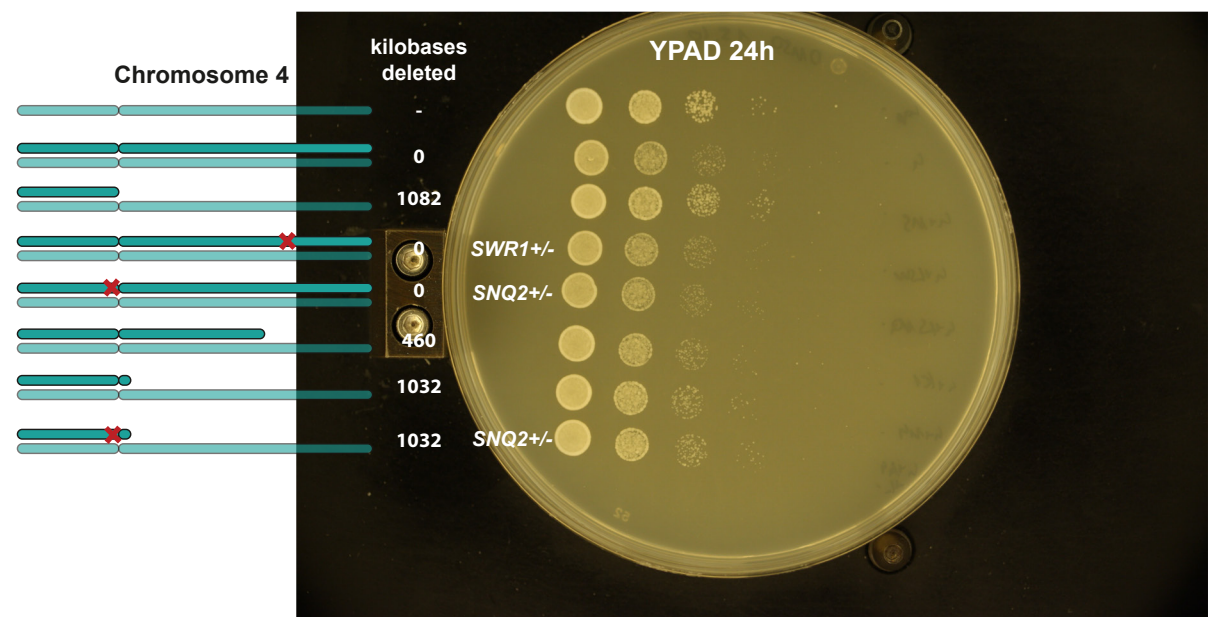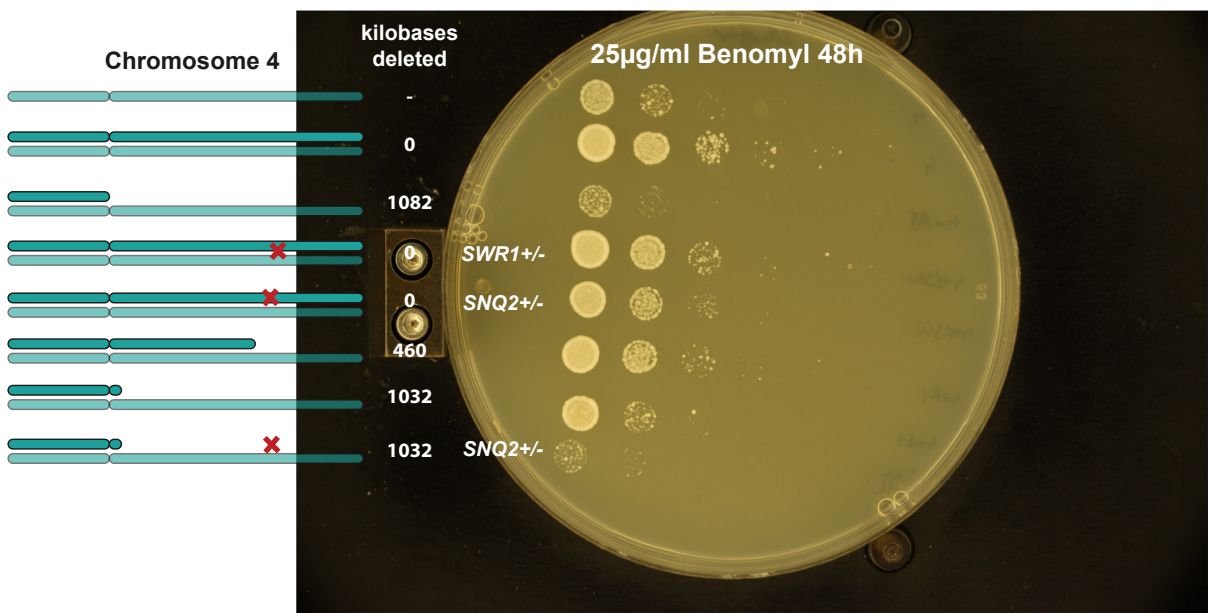

Supplement: Supplementary file 8 — Source data Fig. 5 [file 44319_2026_767_MOESM8_ESM.zip › Figure 5/5B/5B macroscopic photos.pdf]

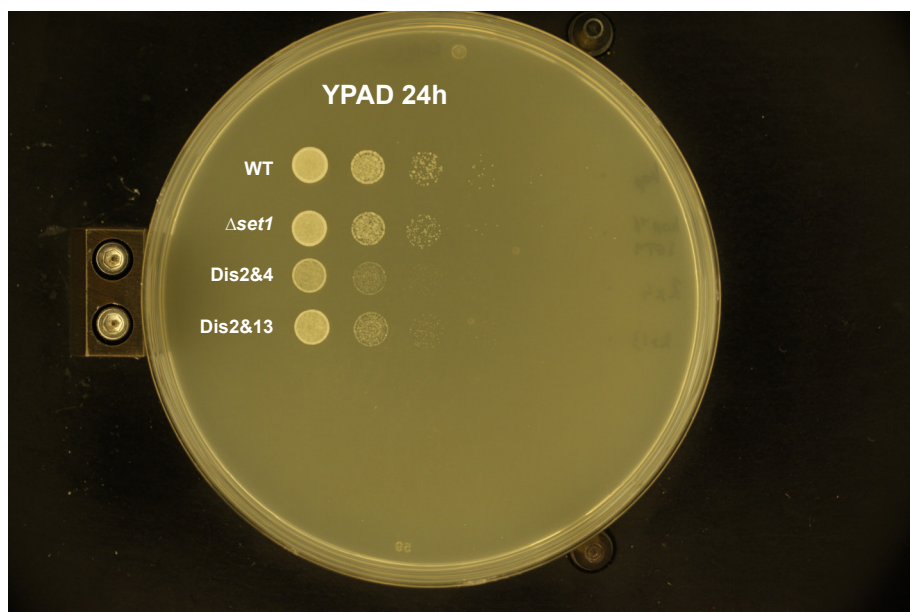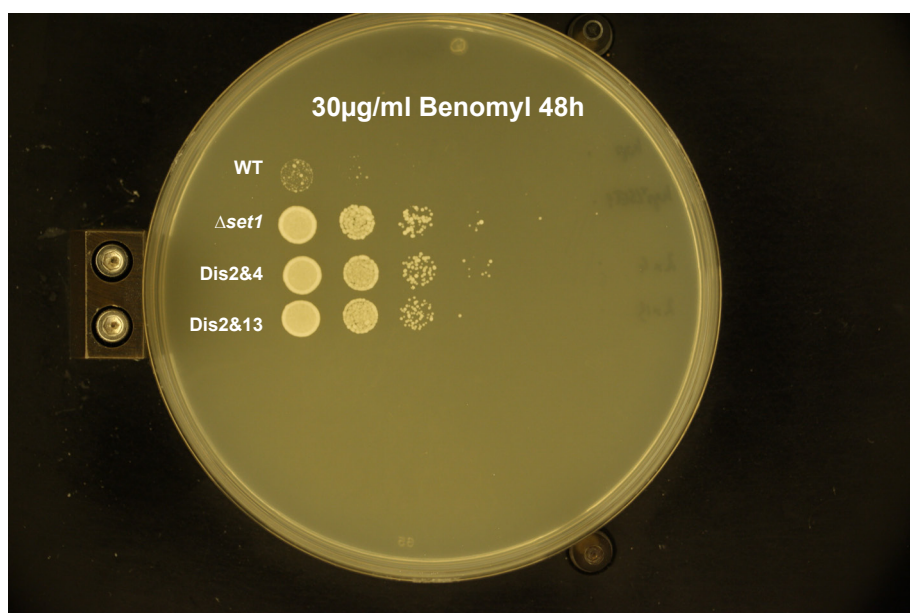

Supplement: Supplementary file 9 — Source data Fig. 6 [file 44319_2026_767_MOESM9_ESM.zip › Figure 6/6C/6C macroscopic photos.pdf]

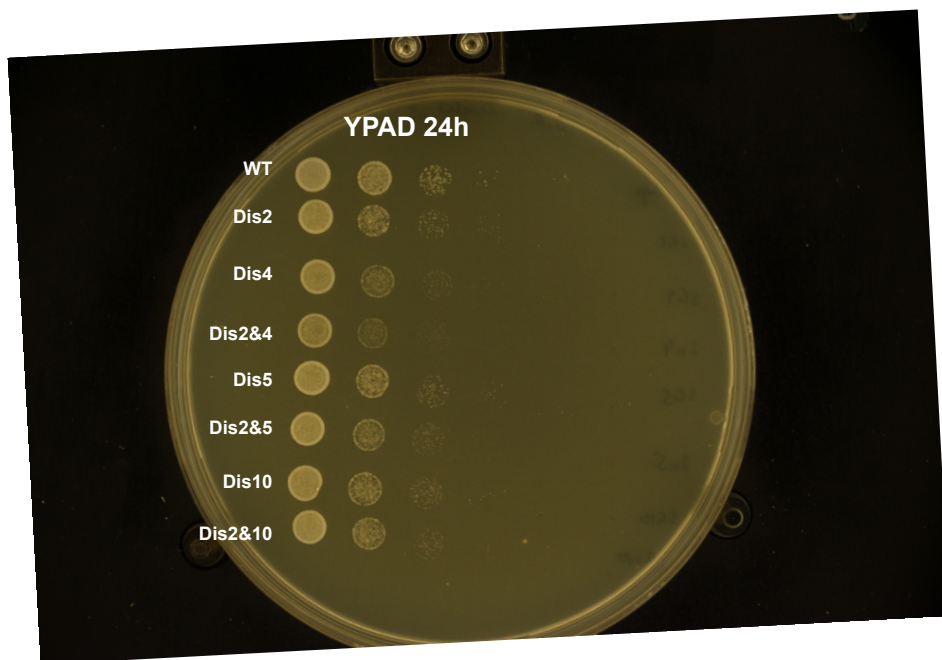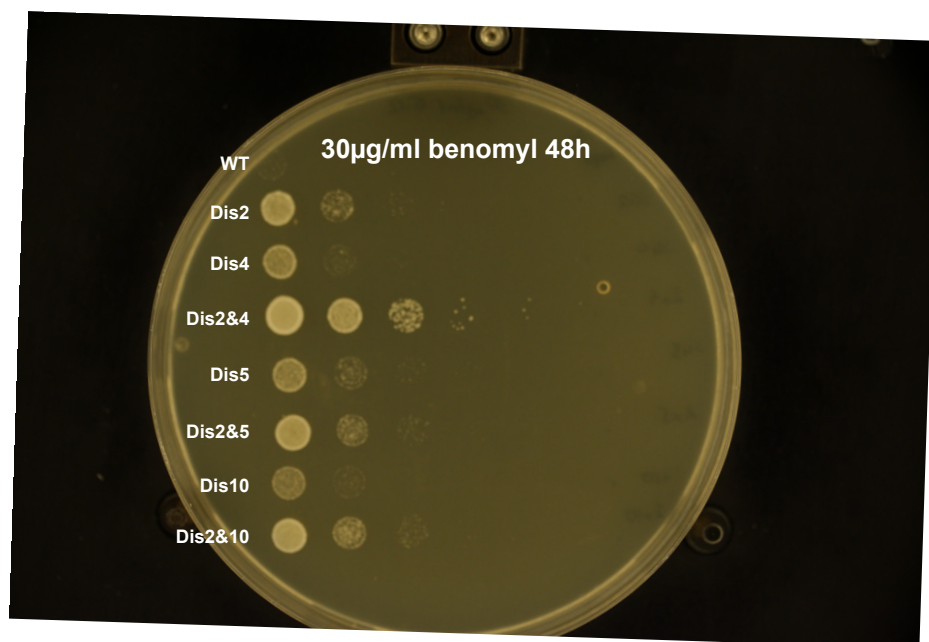

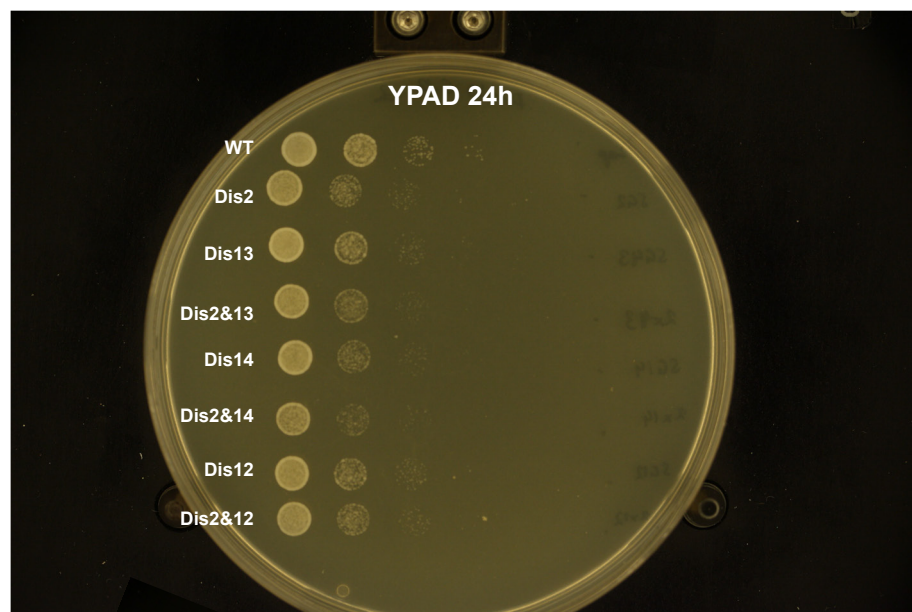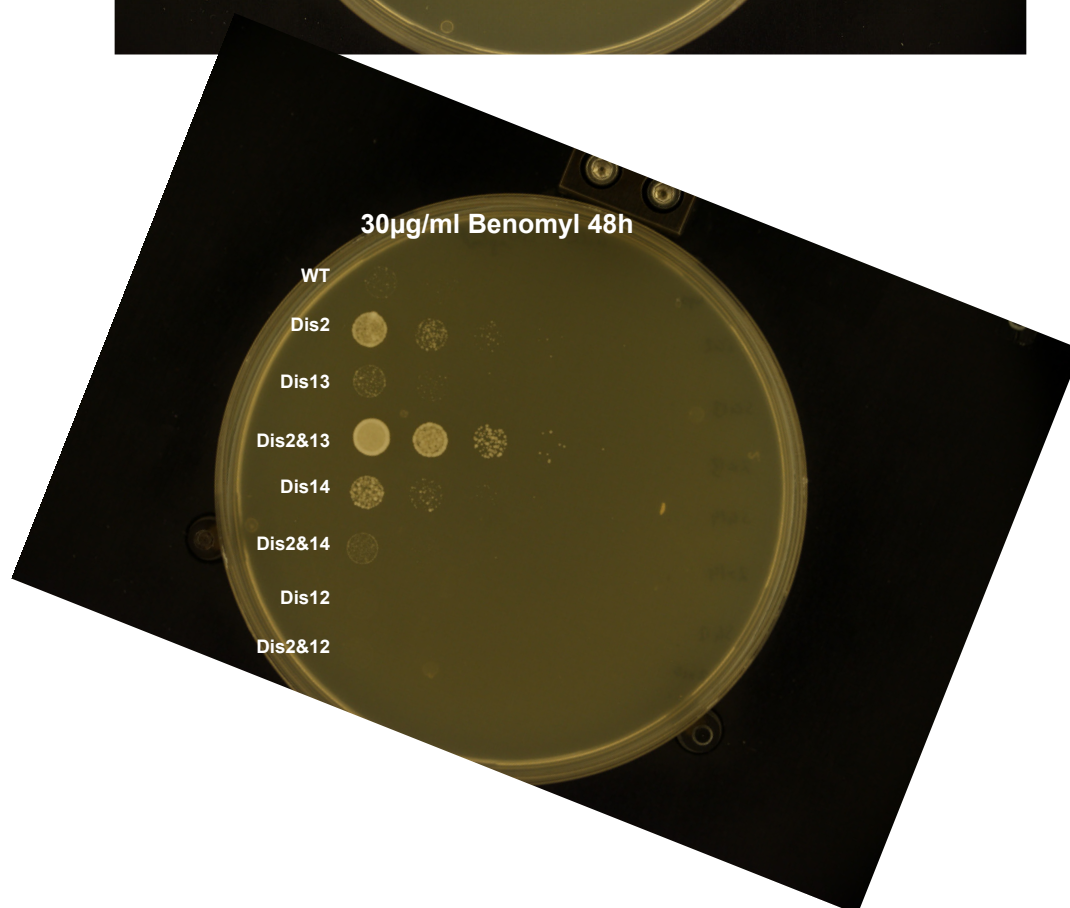

Supplement: Supplementary file 9 — Source data Fig. 6 [file 44319_2026_767_MOESM9_ESM.zip › Figure 6/6B/6B macrsocopic photos.pdf]
