# Supplementary material for: Combinatorial effects of multiple genes contribute to beneficial aneuploidy phenotypes
Source: EMBO Rep. 2026 Apr 11;27(10):2772–97. doi: 10.1038/s44319-026-00767-8 (PMC13219433; doi:10.1038/s44319-026-00767-8)
Supplement: Supplementary file 10 — Expanded View Figures [file 44319_2026_767_MOESM10_ESM.pdf]

Expanded View Figures

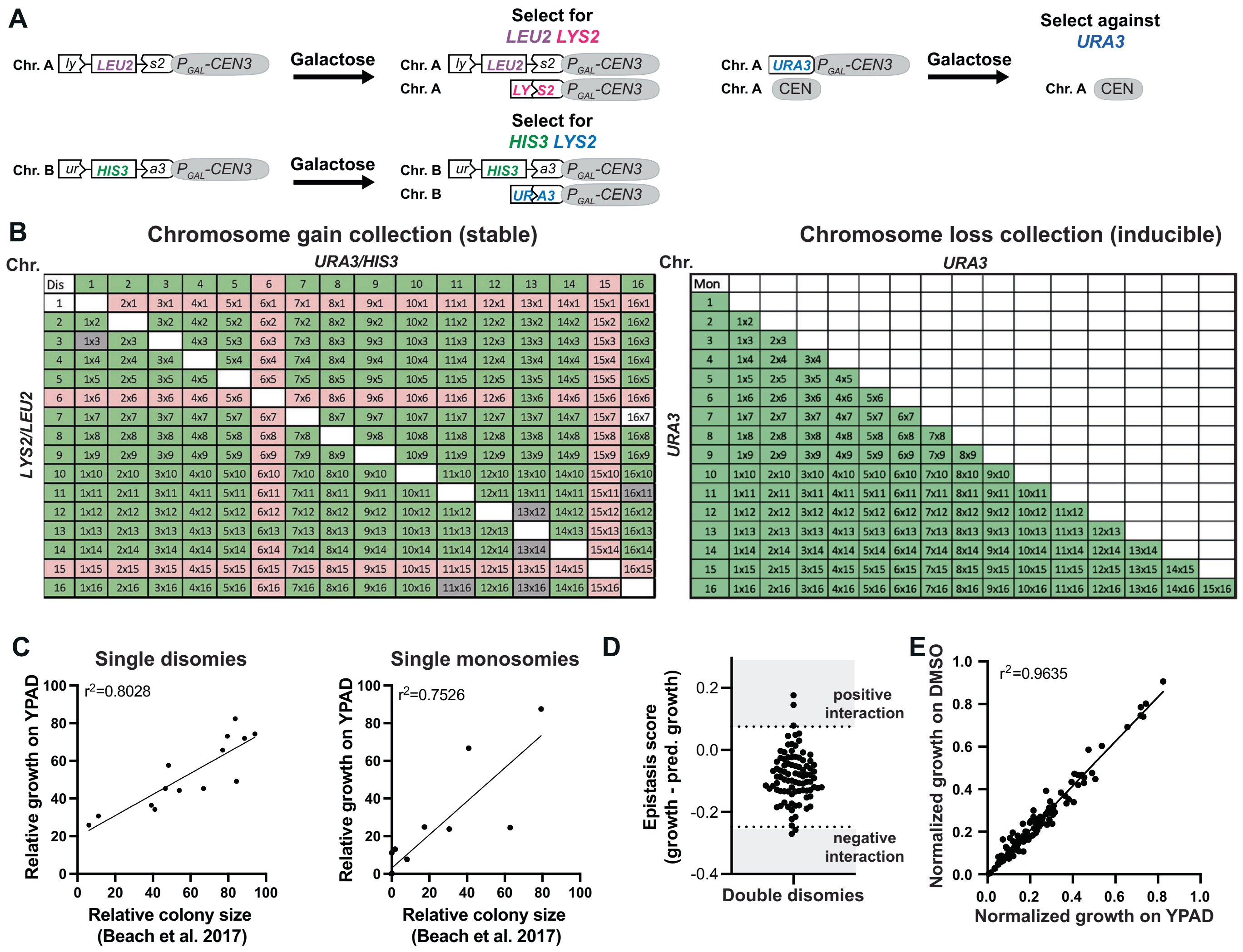

Figure EV1. Engineering of the aneuploid yeast collections.

(**A**) Schematic of the galactose-inducible system for engineering specific disomies and monosomies. Strains were grown in medium containing galactose to induce chromosome nondisjunction during mitosis. Monosomy of the desired chromosome was selected for on plates containing 5-Fluoroorotic Acid (5-FOA), selecting against the URA3 labeled chromosome. Disomy of the desired chromosome was selected for on minimal medium plates lacking leucine, and lysine, or histidine, and uracil, or all four amino acids. (**B**) Overview of the karyotypes represented in the chromosome gain collection (left) and the chromosome loss collection (right). Karyotypes highlighted in green are included in the collection, karyotypes highlighted in gray were excluded from the analysis due to diploidization and those highlighted in red are combinatorial lethal or the euploid parent strain had fitness defects. All possible single monosomies and monosomy combinations are inducible for chromosome loss, but not all are viable. (**C**) Correlation between growth of the single disomies (left) and monosomies (right) from two independent high-throughput spot assays to growth of the single disomies and monosomies from Beach et al, 2017. All strains were normalized to their respective WT controls (disomies: $r^2 = 0.749$, $P < 0.0001$, monosomies: $r^2 = 0.753$, $P < 0.0001$). r^2 values are from simple linear regression, and P value are from F-tests. (**D**) Scatter plot of epistasis scores (measured growth - predicted growth) of the double disomies. Growth predictions are based on the product of the respective single disomies. Kolmogorov-Smirnov test for normality testing was used to test for a normal distribution (KS = 0.05935 and P value > 0.1). Negative and positive interchromosomal interactions are defined by z-scores < −2 (−0.248) and >2 (0.075) and are indicated by dashed lines. (**E**) Correlation between quantified high-throughput spot assays from control plates (YPAD and YPAD + DMSO) ($r^2 = 0.9634$, $P < 0.0001$). r^2 values are from simple linear regression and P value are from F-tests.

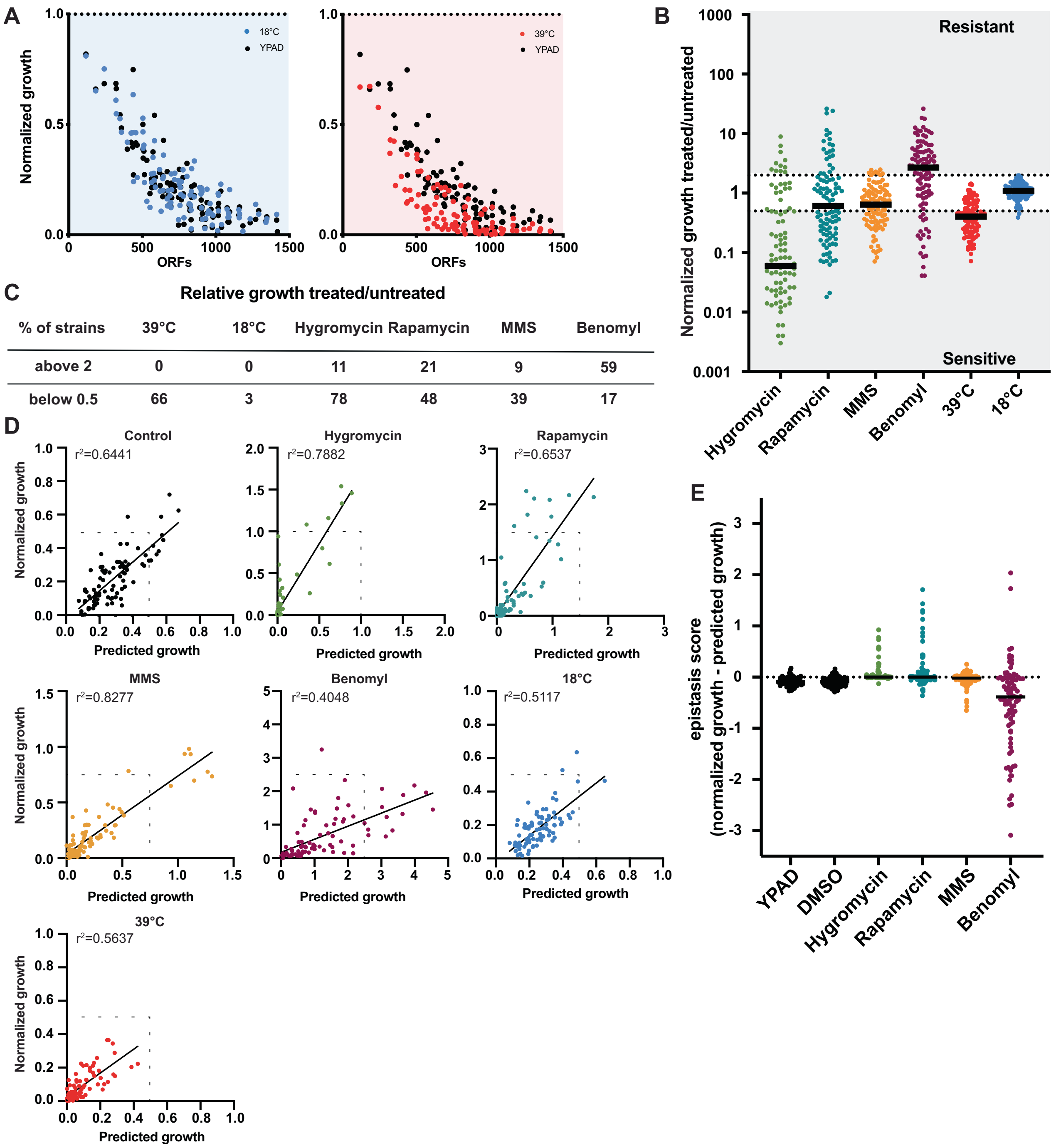

Figure EV2. Genetic interactions between aneuploid chromosomes are more common under selective conditions.

(**A**) Growth of strains from the chromosome gain collection. Control plates were quantified after 24 h (black) and temperature-shifted plates after 30 h (39 °C, red) and 48 h (18 °C, blue). All measurements were from two independent experiments and normalized to a haploid WT. (**B**) Relative growth of the chromosome gain collection under selective conditions normalized to both WT and untreated control plates. Relative growth >2 is counted as resistant, and relative growth <0.5 as sensitive. All measurements were from two independent experiments using 3–6 biological replicates. (**C**) Table of the percentage of strains that are resistant or sensitive to each treatment shown in (**B**). (**D**) Correlations between the growth of double disomies and predicted growth based on single disomies of two independent high-throughput growth assays. Assay conditions: YPAD averaged with YPAD + DMSO (black, $r^2 = 0.6441$, $P < 1.0 \times 10^{-15}$), YPAD + 40 μg/ml hygromycin (green, $r^2 = 0.7882$, $P < 1.0 \times 10^{-15}$), YPAD + 5 nM rapamycin (blue, $r^2 = 0.6537$, $P < 1.0 \times 10^{-15}$), YPAD + 0.02% MMS (orange, $r^2 = 0.8277$, $P < 1.0 \times 10^{-15}$), YPAD + 17.5 μg/ml benomyl (magenta, $r^2 = 0.4048$, $P = 2.08 \times 10^{-11}$), YPAD under cold stress (18 °C, blue, $r^2 = 0.5117$, $P = 3.0 \times 10^{-15}$) and YPAD under heat stress (39 °C, red, $r^2 = 0.5637$, $P < 1.0 \times 10^{-15}$). The dashed line indicates a perfect match between predicted growth and measured growth. r^2 values are from simple linear regression, and P values are from F-tests. (**E**) Epistasis scores under different growth conditions. Negative scores indicate negative genetic interactions and positive scores indicate positive interactions. All measurements were from two independent experiments using 3–6 biological replicates.

© The Author(s)

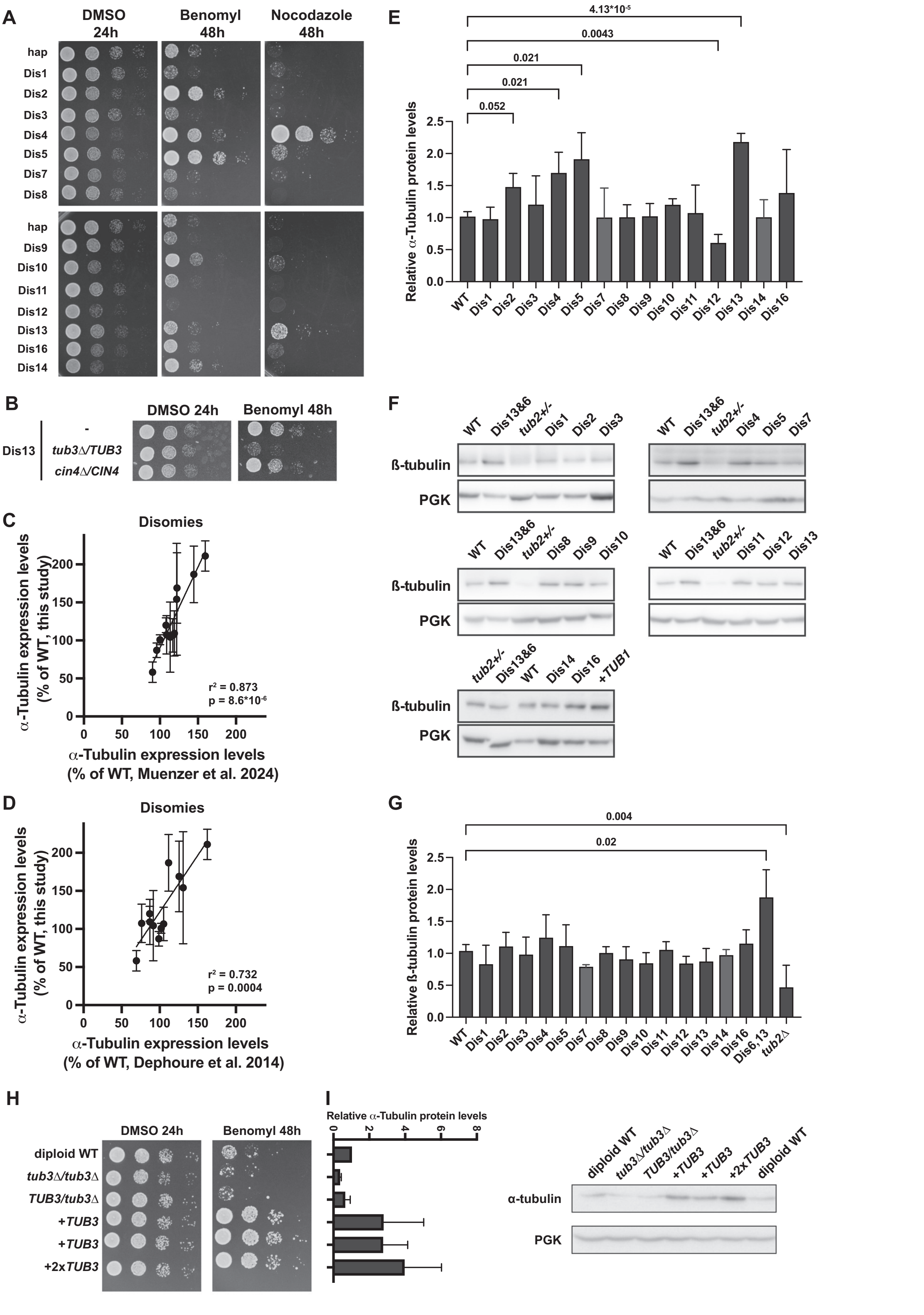

A
DMSO 24h
Benomyl 48h
Nocodazole 48h
hap
Dis1
Dis2
Dis3
Dis4
Dis5
Dis7
Dis8
Dis9
Dis10
Dis11
Dis12
Dis13
Dis16
Dis14
B
Dis13
tub3Δ/TUB3
cin4Δ/CIN4
C
Disomies
α-Tubulin expression levels (% of WT, this study)
α-Tubulin expression levels (% of WT, Muenzer et al. 2024)
r² = 0.873
p = 8.6*10⁻⁶
D
α-Tubulin expression levels (% of WT, Dephoure et al. 2014)
r² = 0.732
p = 0.0004
E
Relative α-Tubulin protein levels
4.13*10⁻⁵
0.0043
0.021
0.021
0.052
F
ß-tubulin
PGK
WT
Dis13&6
tub2+/-
+TUB1
G
Relative ß-tubulin protein levels
0.004
0.02
Dis6,13
tub2Δ
H
diploid WT
tub3Δ/tub3Δ
TUB3/tub3Δ
+TUB3
+2xTUB3
I
Relative α-Tubulin protein levels
α-tubulin
PGK

Figure EV3. Aneuploidy can alter tubulin expression levels independently of tubulin copy numbers.

(**A**) Tenfold serial dilution of all single disomies on agar plates containing DMSO (24 h), 25 µg/ml Benomyl, or 10 µg/ml Nocodazole. Disomies 4, 5, 10 and 13 are resistant to both drugs. (**B**) Tenfold serial dilution of disomy 13, disomy 13 tub3Δ/TUB3, and disomy 13 cin4Δ/CIN4 on YPAD + DMSO (24 h) and YPAD + 20 µg/ml benomyl (48 h) plates. Full serial dilution image including the WT control is in Appendix Fig. S3A. (**C**, **D**) Correlation between α-tubulin expression levels of the single disomies quantified by western blot (Fig. 3B,C, $n = 3$–4, biological) and α-tubulin expression levels of the Torres et al, 2007 single disomies quantified by mass spectrometry. Data from Muenzner et al, 2024 is shown in (**C**) ($P = 8.618 \times 10^{-6)}$ and Dephoure et al, 2014 in (**D**) ($P = 0.0004$). r^2 and P value are calculated from Pearson correlations. Means and standard deviations are shown. (**E**) Quantification of α-tubulin expression levels of the single disomies quantified by western blot (Fig. 3B, $n = 3$, biological). Mean and standard deviation are plotted. P values are calculated using Brown–Forsythe and Welch ANOVA. (**F**) Western blot analysis of β-tubulin expression levels of all single disomies. The double disomy of chromosomes 6 and 13 and a tub2Δ/TUB2 diploid strain were used as controls for changes in β-tubulin expression levels. Pgk1 is shown as a loading control. (**G**) Quantification of ß-tubulin expression levels of the single disomies quantified by western blot ($n = 3$, biological). Mean and standard deviation are plotted. P values are calculated using Brown–Forsythe and Welch ANOVA. (**H**) 10-fold dilution series of diploid strains with different TUB3 copy numbers on YPAD + DMSO (24 h) and YPAD + 15 µg/ml benomyl (48 h) plates. (**I**) Quantification of α-tubulin expression levels via western blot ($n = 3$, biological) for diploid strains with different TUB3 gene copy numbers. Means and standard deviations of three independent experiments are shown. A representative image from one of the blots is shown on the right.

 © The Author(s)

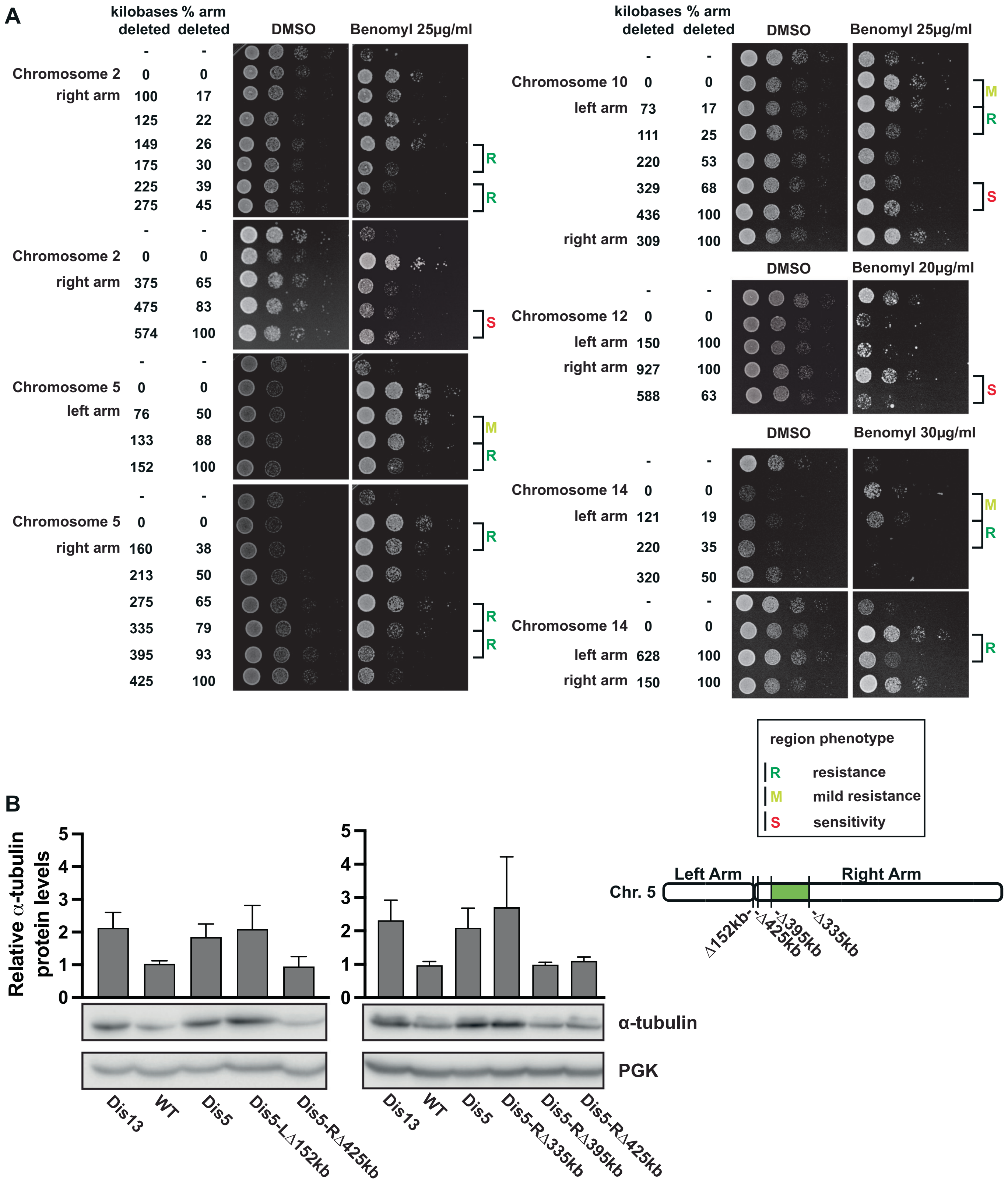
A
kilobases deleted
% arm deleted
DMSO
Benomyl 25µg/ml
Chromosome 2 right arm
Chromosome 5 left arm
Chromosome 5 right arm
Chromosome 10 left arm
right arm
Chromosome 12 left arm
right arm
Benomyl 20µg/ml
Chromosome 14 left arm
right arm
Benomyl 30µg/ml
region phenotype
R resistance
M mild resistance
S sensitivity
B
Relative α-tubulin protein levels
α-tubulin
PGK
Dis13
WT
Dis5
Dis5-LΔ152kb
Dis5-RΔ425kb
Dis5-RΔ335kb
Dis5-RΔ395kb
Chr. 5
Left Arm
Right Arm
Δ152kb
Δ425kb
Δ395kb
Δ335kb

Figure EV4. Multiple regions on aneuploid chromosomes contribute to benomyl resistance.

(**A**) Tenfold serial dilutions of partial chromosome arm deletions for chromosomes 2, 4, 5, 10, 12, and 14. Control plates (YPAD + DMSO) were imaged after 24 h, and treated plates (YPAD + benomyl) after 48 h. (**B**) Quantification of α-tubulin expression levels of the single disomies quantified by western blot ($n = 3$, biological). Dis13 is a control for elevated α-tubulin levels. Mean and standard deviation are plotted. Quantified bands were normalized to the Ponceau-stained membrane and the euploid WT. The diagram on the right shows the location of the cuts on the right and left arms of chromosome 5. The region leading to α-tubulin overexpression is in green.

 © The Author(s)

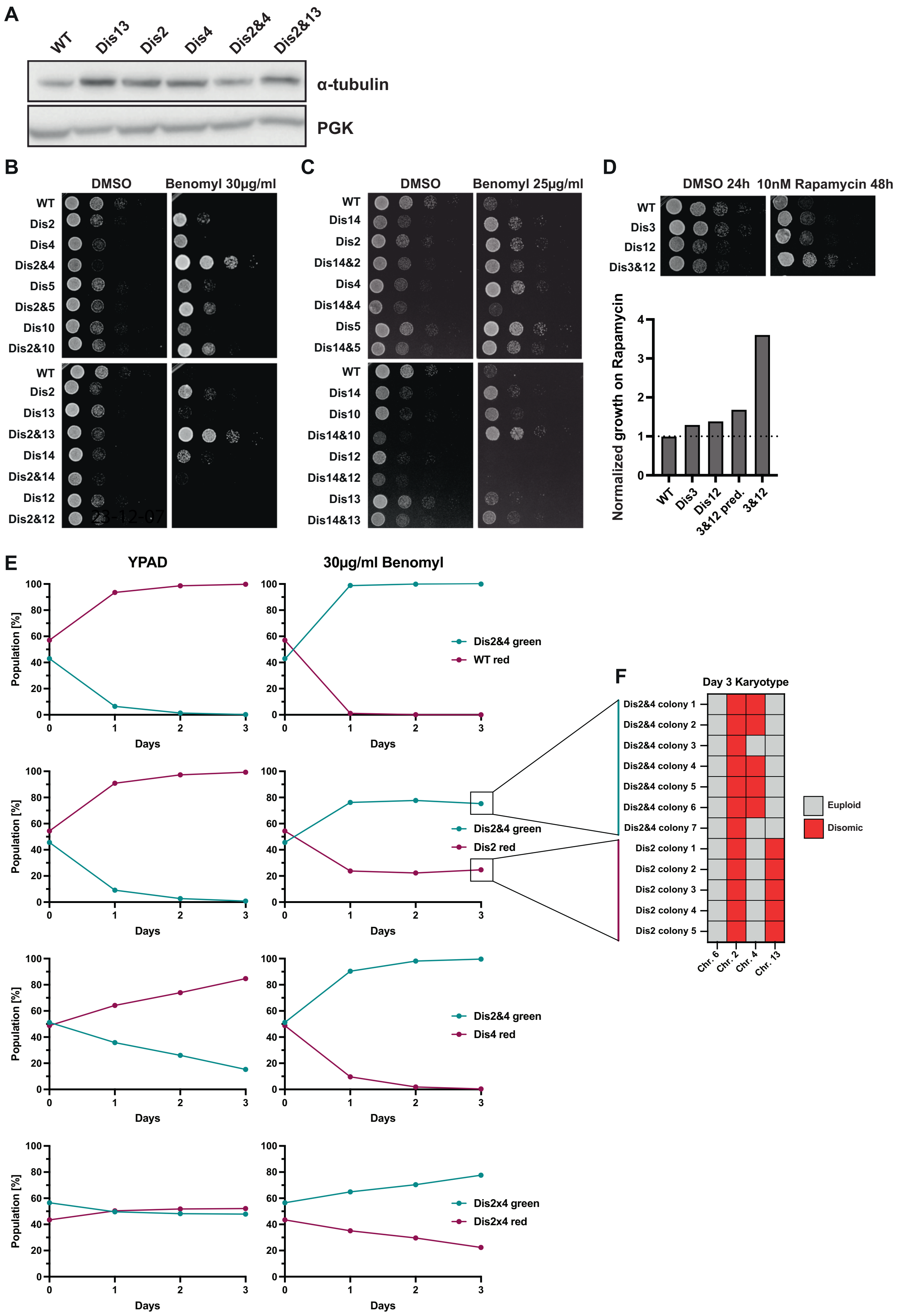
A
WT
Dis13
Dis2
Dis4
Dis2&4
Dis2&13
α-tubulin
PGK
B
DMSO
Benomyl 30µg/ml
WT
Dis2
Dis4
Dis2&4
Dis5
Dis2&5
Dis10
Dis2&10
WT
Dis2
Dis13
Dis2&13
Dis14
Dis2&14
Dis12
Dis2&12
C
DMSO
Benomyl 25µg/ml
WT
Dis14
Dis2
Dis14&2
Dis4
Dis14&4
Dis5
Dis14&5
WT
Dis14
Dis10
Dis14&10
Dis12
Dis14&12
Dis13
Dis14&13
D
DMSO 24h
10nM Rapamycin 48h
WT
Dis3
Dis12
Dis3&12
Normalized growth on Rapamycin
WT
Dis3
Dis12
3&12 pred.
3&12
E
YPAD
30µg/ml Benomyl
Population [%]
Days
Dis2&4 green
WT red
Dis2&4 green
Dis2 red
Dis2&4 green
Dis4 red
Dis2x4 green
Dis2x4 red
F
Day 3 Karyotype
Dis2&4 colony 1
Dis2&4 colony 2
Dis2&4 colony 3
Dis2&4 colony 4
Dis2&4 colony 5
Dis2&4 colony 6
Dis2&4 colony 7
Dis2 colony 1
Dis2 colony 2
Dis2 colony 3
Dis2 colony 4
Dis2 colony 5
Chr. 6
Chr. 2
Chr. 4
Chr. 13
Euploid
Disomic

Figure EV5. Complex aneuploidy outcompetes single aneuploidies under drug selection.

(**A**) Western blot analysis of α-tubulin expression levels for the indicated single and double disomies. (**B**) Tenfold serial dilutions of disomy combinations with chromosome 2. Control plates (YPAD + DMSO) were imaged after 24 h, and treated plates (YPAD + 30 μg/ml benomyl) after 48 h. (**C**) Tenfold serial dilutions of disomy combinations with chromosome 14. Control plates (YPAD + DMSO) were imaged after 24 h, and treated plates (YPAD + 25 μg/ml benomyl) after 48 h. (**D**) Tenfold serial dilutions and quantifications of disomy 3 and 12 on control (YPAD + DMSO) and treated (10 nM rapamycin) plates. All quantifications were normalized to a haploid WT. Combinatorial growth of chromosomes 3 and 12 double disomy was predicted by adding the phenotypes of the single disomies. (**E**) Liquid culture competition assay between different strains in control (YPAD + DMSO) and selective (YPAD + 30 μg/ml Benomyl) conditions. Cultures were diluted into fresh media every day, and samples were fixed with PFA and analyzed using flow cytometry. (**F**) qPCR measurements of chromosome copy numbers from single colonies from the 3rd day of the competition between Dis2 and Dis2&4 in selective (YPAD + 30 μg/ml Benomyl) conditions.

 © The Author(s)
